# Comparative analyses of glycerotoxin expression unveil a novel structural organization of the bloodworm venom system

**DOI:** 10.1186/s12862-017-0904-4

**Published:** 2017-03-04

**Authors:** Sandy Richter, Conrad Helm, Frederic A. Meunier, Lars Hering, Lahcen I. Campbell, Stephan H. Drukewitz, Eivind A. B. Undheim, Ronald A. Jenner, Giampietro Schiavo, Christoph Bleidorn

**Affiliations:** 1 Institute of Biology - Molecular Evolution and Systematics of Animals, University of Leipzig, Talstraße 33, D-04103 Leipzig, Germany; 2 German Centre for Integrative Biodiversity Research (iDiv) Halle-Jena-Leipzig, Deutscher Platz 5e, D-04103 Leipzig, Germany; 3 Department of Life Sciences, Natural History Museum, Cromwell Rd, London, SW7 5BD UK; 4 Sars International Centre for Marine Molecular Biology, University of Bergen, Thormøhlensgate 55, N-5008 Bergen, Norway; 5 Clem Jones Centre for Ageing Dementia Research, Queensland Brain Institute, University of Queensland, St. Lucia, Brisbane, 4072 QLD Australia; 6 Institute of Biology - Department of Zoology, University of Kassel, Heinrich-Plett-Straße 40, D-34132 Kassel, Germany; 7 The European Bioinformatics Institute (EMBL-EBI) - Wellcome Genome Campus, Hinxton, Cambridge, CB10 1SD UK; 8 Centre for Advanced Imaging, University of Queensland, St. Lucia, Brisbane, 4072 QLD Australia; 9 Sobell Department of Motor Neuroscience & Movement Disorders, UCL Institute of Neurology, University College London, Queen Square, London, WC1N 3BG UK; 10 Museo Nacional de Ciencias Naturales, Spanish National Research Council (CSIC), Calle José Gutierrez Abascal 2, 28006 Madrid, Spain

**Keywords:** Annelida, Glyceridae, Calcium channel, Neurotoxin, GLTx, Venom system

## Abstract

**Background:**

We present the first molecular characterization of glycerotoxin (GLTx), a potent neurotoxin found in the venom of the bloodworm *Glycera tridactyla* (Glyceridae, Annelida). Within the animal kingdom, GLTx shows a unique mode of action as it can specifically up-regulate the activity of Ca_v_2.2 channels (N-type) in a reversible manner. The lack of sequence information has so far hampered a detailed understanding of its mode of action.

**Results:**

Our analyses reveal three ~3.8 kb GLTx full-length transcripts, show that GLTx represents a multigene family, and suggest it functions as a dimer. An integrative approach using transcriptomics, quantitative real-time PCR, *in situ* hybridization, and immunocytochemistry shows that GLTx is highly expressed exclusively in four pharyngeal lobes, a previously unrecognized part of the venom apparatus.

**Conclusions:**

Our results overturn a century old textbook view on the glycerid venom system, suggesting that it is anatomically and functionally much more complex than previously thought. The herein presented GLTx sequence information constitutes an important step towards the establishment of GLTx as a versatile tool to understand the mechanism of synaptic function, as well as the mode of action of this novel neurotoxin.

**Electronic supplementary material:**

The online version of this article (doi:10.1186/s12862-017-0904-4) contains supplementary material, which is available to authorized users.

## Background

Complex venoms and associated venom systems have independently evolved in a broad phylogenetic range of animals where they play a diversity of biological roles including defense, competition and predation [1–3]. Despite their similar usage, highly diverse venom systems with remarkable variation evolved not only between different venomous clades but also within single clades [1]. Potent neurotoxins, which act as modulators on a variety of ion channels, are a conspicuous component of many venom cocktails with proven pharmacological and therapeutic potential [4]. One specific venom peptide, ω-conotoxin GVIA, from marine snails of the genus *Conus*, is a widely used pharmacological tool in neuroscience because of its ability to block Ca_v_2.2 (N-type) calcium channels [5, 6]. Another blocker of this channel, ω-conotoxin MVIIA, isolated from *Conus magus*, was the first FDA-approved drug for intractable chronic pain [7]. Calcium channels regulate the permeability of cell membranes to calcium ions (Ca^2+^) in a voltage-controlled manner, and they function as key transducers for the intracellular flow of calcium [4, 8]. Ca^2+^ is in turn of critical importance for the regulation of many biological processes in eukaryotic cells. In neurons, Ca^2+^ is instrumental for the transmission of nerve signals and synaptic activity [9] as its entry leads to fusion of synaptic vesicles and subsequent neurotransmitter release into the synaptic cleft [10]. Calcium channel blockers have been isolated from various animal venoms such as snakes, spiders and mollusks [2]. In contrast, there are very few accounts of calcium channel activators within animal venoms. A voltage-gated calcium channel (Ca_v_) agonist has been described from centipede venom (ω-SLPTX-Ssm1a) in the genus *Scolopendra*, although its mode of action and target specifity remain unknown [11, 12]. Agonists of Ca_v_ sub-type 2.2 (N-type) are particularly rare [2], with glycerotoxin, which was purified from a marine annelid of the genus *Glycera*, being the best example [13].

Glycerotoxin-producing polychaetes belong to the taxon Glyceridae Grube, 1850 (Annelida), and are commonly known as bloodworms. These venomous annelids show a broad worldwide distribution and are easily recognizable by an eversible pharynx that possesses four cross-arranged teeth consisting of a hook-shaped jaw and an aileron (supporting structure), each of which is associated with a putative venom gland [14–21]. The putative venom glands are each surrounded by massive layers of musculature [22] whose contraction plays an essential role in the process of envenomation [17]. These glands are further connected by special ducts to the teeth that exhibit a series of pores through which the venom is delivered [14, 17, 23]. Furthermore, biomineralization with the copper-based biomineral atacamite [Cu_2_(OH)_3_Cl] enhances the stiffness and hardness of the jaws and makes them remarkably resistant to abrasion. Beyond its mechanical function, it is speculated that copper may play a role in the activation of venom during injection [24].

The existence of a venom apparatus strongly suggested the presence and utilization of venom in Glyceridae. Further insights were gained through proteomic studies on purified fractions of the venom gland cocktail of *Glycera tridactyla* (formerly *G. convoluta*). Michel, Keil [25] recovered low and high molecular weight toxins, and further demonstrated a paralytic function of the latter. Subsequent studies revealed the ability of a high-molecular weight component to reversibly increase spontaneous neurotransmitter release [26, 27]. This neurotoxic activity of *G. tridactyla* venom correlated with the presence of a 300–320 kDa glycoprotein [13, 22, 28], known as glycerotoxin (GLTx). This neurotoxin was found to target Ca_v_2.2 channels (N-type), which are expressed in the presynaptic plasma membrane, and causes an increased Ca^2+^ influx at resting potential [13]. By its ability to specifically up-regulate the activity of Ca_v_2.2 channels, GLTx follows a so far unique mode of action. As a result, this neurotoxin dramatically increases neurotransmitter release in a variety of preparations [13, 29], which makes it a versatile tool to analyze the physiological mechanisms affecting neuroexocytosis. Furthermore, GLTx is able to up-regulate the process of presynaptic vesicle recycling at the frog neuromuscular junction and therefore has been pivotal to secure a detailed understanding of the mechanism of bulk endocytosis at nerve terminals [30]. However, whereas the functional properties of glycerotoxin are well-described, missing sequence data and poor anatomical characterization of the venom apparatus have hampered understanding of the mode of envenomation and actual mode of action on Ca^2+^ channels.

In our study, we performed the first molecular characterization of glycerotoxin in the bloodworm species *G. tridactyla*. We followed a multidisciplinary approach combining transcriptomic and proteomic analyses, qPCR experiments, *in situ* hybridization, antibody staining and bioinformatic analyses. Our studies elucidated three full-length transcripts of GLTx, and provided new insights into its mechanisms of action and evolution. Moreover, our integrated approach led to the discovery of previously unrecognized pharyngeal toxin-producing structures, demonstrating that the glycerid venom apparatus is anatomically and functionally more complex than previously thought. This unexpected complexity provides a step change in our understanding of the structure of the venom system in glycerid annelids, breaking a century old consensus in the field. Furthermore, the first cloning and sequence analysis of GLTx pave the way for more in-depth analyses of this unique neurotoxin capable of stimulating neuronal communication.

## Results

### Characterization of glycerotoxin (GLTx)

Edman sequencing of purified glycerotoxin (GLTx) yielded a series of short amino acid sequences varying in length between 7–18 amino acids (Additional file 1). Using them as reference sequences in BLAST-searches against transcriptome data led to the identification of GLTx in *G. tridactyla*. In the transcriptome library of the pharyngeal lobes (SR23, single specimen), we were able to recover three full-length transcripts of GLTx. In contrast, the putative venom glands (SR21, single specimen and SR22, pooled multiple specimen) exhibited only a few fragments of the GLTx full-length transcript, whilst in the body tissues (SR25, single specimen and SR26, pooled multiple specimen) we were not able to identify any GLTx transcripts.

Cloning of the nearly full-length GLTx gene (length ~3600 bp) amplified from cDNA (using SR23, contig 5772 as reference) and subsequent primer walking revealed the nucleotide sequence of glycerotoxin (Additional file 2). The cloned sequences are concordant in length to the recovered GLTx full-length transcript from the transcriptome assembly (SR23, contig 5772). The highly similar clones 5A and 7 each harbor a 12 bp insertion which is missing in clone 6A. Translations of obtained GLTx sequences contain an unexpected stop codon at the 5’-end of clone 5A (Additional file 3). Two further GLTx full-length transcripts (SR23, contig 4317 and contig 4318) are similar to each other but remarkably different from contig 5772. They presumably represent a different GLTx paralog (Additional file 3).

Without a signal peptide, the translated GLTx full-length transcript (SR23, contig 5772) has a length of 1257 amino acids, and the two full-length transcripts (SR23, contig 4317 and contig 4318) have a length of 1256 amino acids. According to a size estimation performed in CLC Main Workbench, the molecular weight of the proteins deduced from the three GLTx full-length transcripts is around 140 kDa each. SDS-PAGE of *G. tridactyla* venom followed by in-gel digestion and identification by tandem mass spectrometry concordantly revealed a molecular weight of the complete GLTx polypeptide chain of around 150 kDa (Additional file 4: Figure S1 and Additional file 5). The 320 kDa band which correlates with the activity of GLTx could not be recovered.

**Fig. 1:**
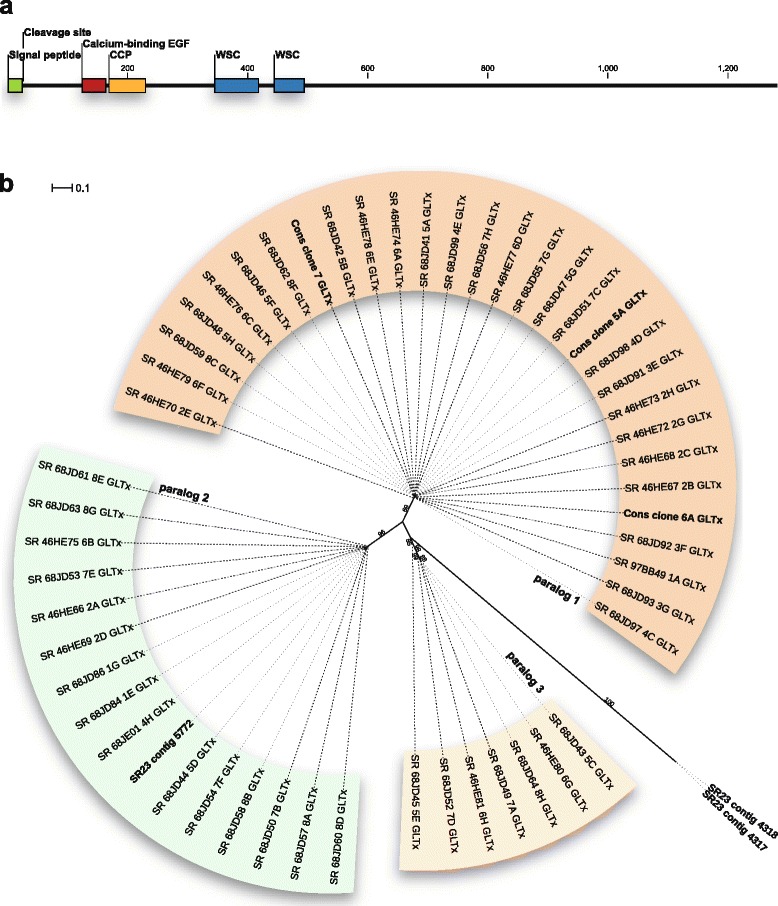
Molecular characterization of the glycerotoxin gene analyzed in the bloodworm species *G. tridactyla* (Glyceridae, Annelida). **a** Domains identified in three translated GLTx full-length transcripts (SR23, contig 5772, contig 4317, and contig 4318). A calcium-binding EGF domain and two WSC domains were recovered by Pfam-searches, and a CCP domain through SMART. The full-length transcripts each harbor a signal peptide, but no known protein domains are present at the C-terminal end of the GLTx gene. **b** Maximum likelihood analysis performed on a dataset comprising a 746 bp gene fragment of 52 GLTx clones and three GLTx full-length transcripts (SR23, contig 5772, contig 4317, and contig 4318) revealed at least three paralogs, namely GLTx paralog 1, GLTx paralog 2, and GLTx paralog 3. The ML phylogeny obtained with RAxML v.8.2.8 represents the best tree under a GTR + GAMMA + I substitution model. Bootstrap support values (>60%) from 1,000 pseudoreplicates are given at the nodes. Scale bars indicate the number of substitutions per site

In all analyzed GLTx transcripts, protein domain searches yielded a calcium-binding EGF domain, two WSC domains (cell wall integrity and stress response component), and a CCP domain (complement control protein, also known as short consensus repeats SCRs or sushi repeats), all of which are found at the N-terminal end of the protein (Fig. 1a). The computationally determined full-length transcripts (SR23, contig 5772, contig 4317, and contig 4318) each have a signal peptide (Fig. 1a). However, no known protein domains could be identified within the C-terminal end of GLTx. Our data reveals that GLTx is a unique neurotoxin with 80% of its sequence displaying a completely unknown domain organization. BLAST-searches of GLTx clones and GLTx full-length transcripts in NCBI GenBank yielded hits to an uncharacterized protein in *Branchiostoma*, and a collectin-12 sequence in *Exaiptasia pallida* (Cnidaria, KXJ16027.1). Moreover, sequence matches were found in expressed sequence tags (ESTs) of the annelids *Myzostoma cirriferum* (FN428144.1) and *Pomatoceros lamarckii* (GR311097.1). A maximum likelihood phylogenetic analysis on 52 clones shows the presence of at least three paralogs, named GLTx paralog 1, paralog 2, and paralog 3 (Fig. 1b and Additional file 6).

PCR experiments using genomic DNA revealed at least four introns (Additional file 4: Figure S2 and Additional file 7). We focused mainly on the intron-exon-structure at the 3’-end of the gene, especially regions adjacent to the exon 3 analyzed in the context of qPCR studies and *in situ* hybridization experiments. The intron-exon-structure at the 5’-end (exon 1) remains unknown.

### GLTx localization within the glycerid venom apparatus

**Fig. 2:**
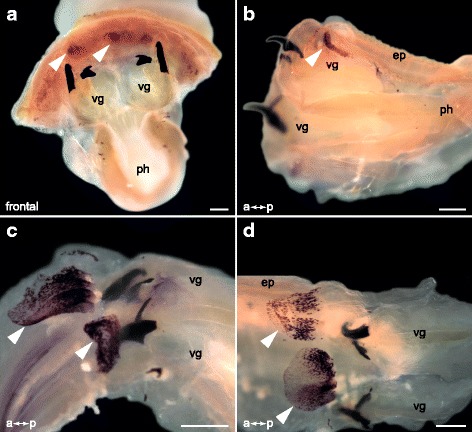
Expression of GLTx in bisected pharynges of adult *G. tridactyla*. Stereomicrographs. Arrows indicate *in situ* hybridization signal. **a–b** Frontal and lateral view on a glycerid pharynx cut into two halves. GLTx expression is restricted to lobate structures that are attached to the wall of the proboscis near the base of the teeth, and is absent from any other pharyngeal tissues. **c–d** GLTx expression occurs in clearly defined pharyngeal lobes. ep, epithelium; ph, pharynx; vg, putative venom gland. Scale bars: 100 μm (**a–d**)

We next aimed to identify the site of toxin expression. We performed *in situ* hybridization experiments with digoxigenin-labeled RNA probes on everted glycerid pharynges, which were dissected into two halves. Prominent GLTx expression is detected in lobate structures located near the base of the teeth, attached to the wall of the proboscis (Fig. 2a,b). Expression signal is restricted to these clearly defined pharyngeal lobes (Fig. 2c,d), and to tissue located at the base of the teeth (Fig. 4b). No other distinct expression was visible in the putative venom glands or elsewhere in the pharynx (Fig. 2). The basal parts of the lobes show strong GLTx expression, whereas the distal parts exhibit a fainter signal (Fig. 2c,d).

**Fig. 3:**
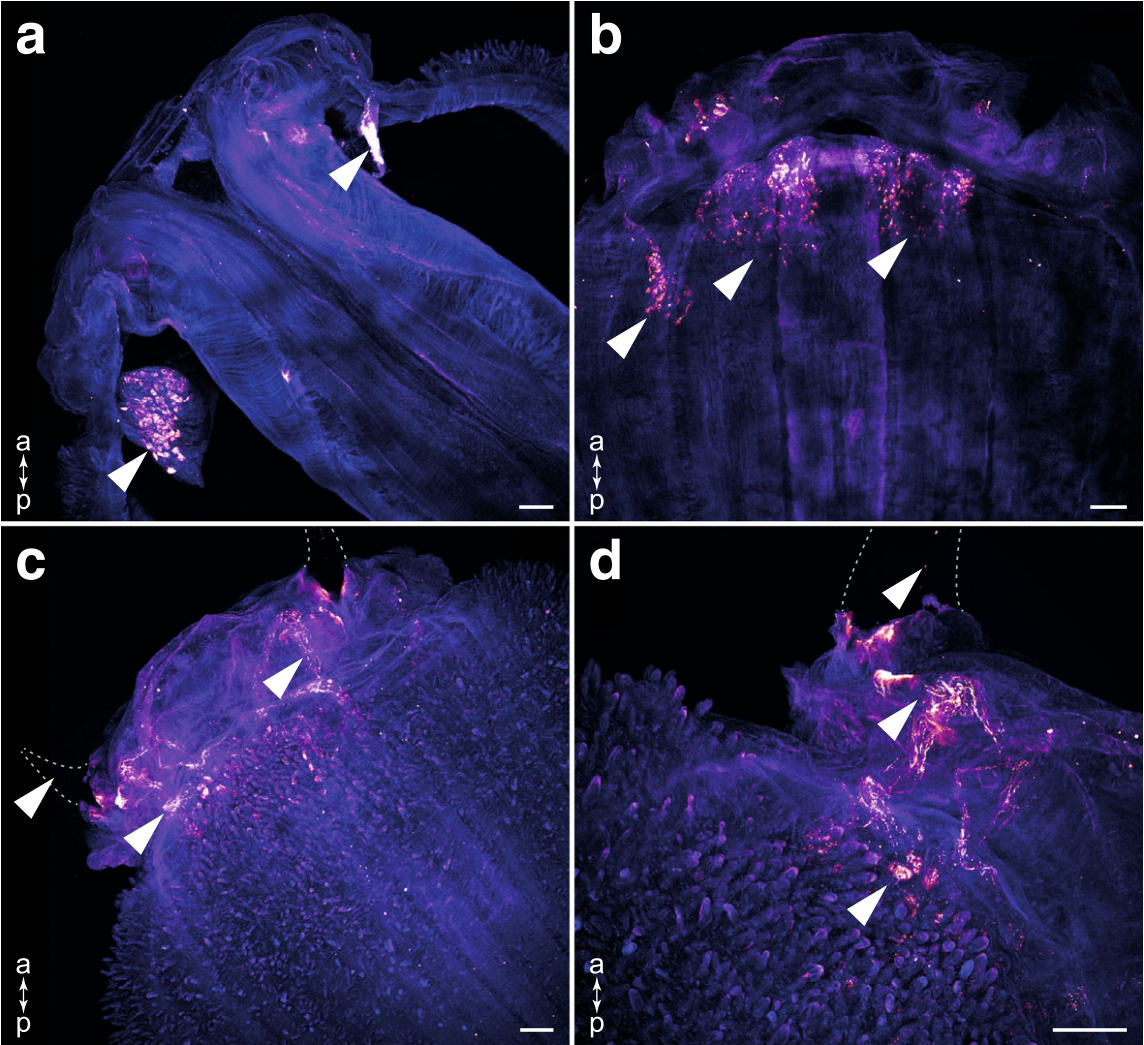
Confocal maximum projections of everted *G. tridactyla* pharynges cut into two halves. Anti-GLTx staining (glow-mode) and phalloidin–rhodamine counterstaining (blue). Arrows indicate GLTx-IR staining. **a** Distinct GLTx-IR staining occurs in clearly defined pharyngeal lobes. Note there is no additional staining inside other pharyngeal tissues. **b** Anti-GLTx staining revealed a radial color pattern that is most prominent at the base and appears faint in apical parts of the lobes. **c–d** A network of duct-like structures (which we refer to as ducts) connects and transports the GLTx from the pharyngeal lobes to the teeth. Through a series of pores the venom is delivered. Scale bars: 100 μm (**a–d**)

**Fig. 4:**
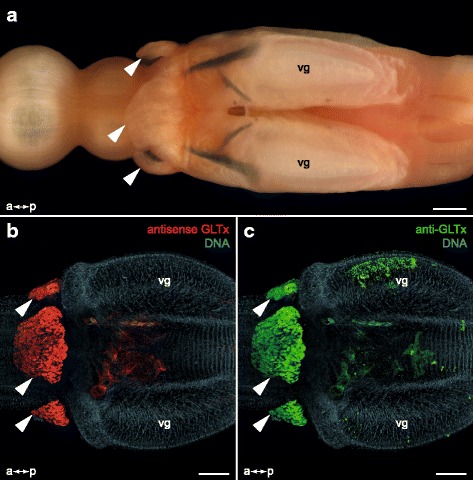
Confocal maximum projections of an inverted pharynx of *G. tridactyla* analyzed as total. Fluorescence *in situ* hybridization (FISH) coupled with antibody staining against GLTx, and TO-PRO®-3 Iodide counterstaining. **a** Overview of an inverted glycerid pharynx comprising four cross arranged putative venom glands each connected to a tooth, and four corresponding pharyngeal lobes (three of them marked by an arrow). **b** Fluorescence *in situ* hybridization (FISH) revealed a clear GLTx signal in the lobes (marked by arrows) and tissue at the base of the teeth. Note that there is no distinct staining visible in the putative venom glands. **c** Distinct GLTx-IR staining (marked by arrows) is solely restricted to the pharyngeal lobes. Note that there is no staining signal inside the putative venom glands or additional pharynx tissues. vg, putative venom gland. Scale bars: 100 μm (**a–c**)

**Fig. 5:**
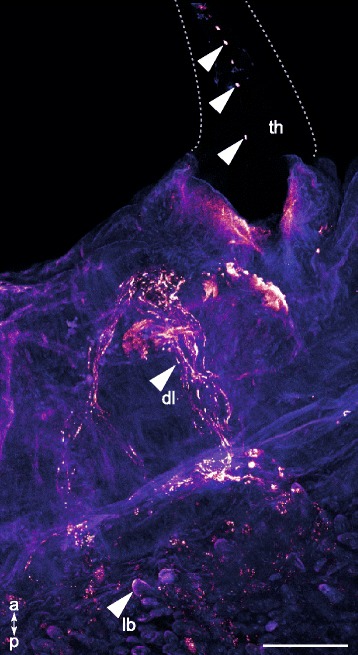
Immunolocalization of GLTx in a bisected *G. tridactyla* pharynx. Confocal maximum projection. Arrows indicate GLTx-IR staining. Anti-GLTx staining is restricted to clear defined lobate structures. From these pharyngeal lobes, the GLTx is transported through a network of duct-like structures (which we refer to as ducts) directly to the teeth, and squeezed out through a series of pores. dl, duct-like structures; lb, part of the lobes; th, teeth. Scale bar: 100 μm


To identify pharynx components presumably responsible for GLTx storage, we performed further anti-GLTx antibody staining on everted glycerid pharynx samples also equally dissected into two halves. Distinct anti-GLTx-immunoreactivity (GLTx-IR) is visible in the clearly defined pharyngeal lobes (Fig. 3a), and is most prominent at the base and appears fainter in distal parts of the lobe (Fig. 3b–d). Moreover, fluorescence *in situ* hybridization (FISH) coupled with antibody staining against GLTx reveals that neurotoxin expression and storage is restricted to the same set of cells within the lobe (Fig. 4), indicating that these cells express and secrete GLTx. The double-staining approach performed on an entire inverted pharynx revealed altogether four pharyngeal lobes, each one associated with a single tooth. Additional GLTx-IR staining outside the pharyngeal lobes was not detectable when analyzing pharynx samples that were not embedded (Fig. 3a and Fig. 4c). Notably, analyses on paraffin embedded cross sections of the putative venom glands revealed a prominent GLTx-IR signal inside the lumen (Additional file 4: Figure S3). These results agree with previous work in which GLTx was identified through activity tests in venom fractions extracted from putative venom glands [13, 22, 28]. In what extent the putative venom glands are involved in the storage of GLTx as well as whether there are ducts connecting the pharyngeal lobes and putative venom glands remain to be further investigated. However, antibody staining against GLTx unveils a network of duct-like structures (which we herein refer to as ducts) leading from the lobes to the associated tooth (Fig. 3c,d). It therefore seems that both pharyngeal lobes and putative venom glands are directly connected to the teeth. GLTx-IR also showed that GLTx is released through a series of pores on the teeth (Fig. 5 and Additional file 8).

Additional file 8:Immunolocalization of GLTx in a pharynx of *G. tridactyla* which was cut into two halves. Confocal maximum projection. Anti-GLTx staining occurs in clear defined pharyngeal lobes. GLTx-IR staining revealed a canalized GLTx transport from the lobes to the teeth, where it becomes delivered through a series of pores. (AVI 117177 kb)


To analyze whether the lobes of the glycerid pharynx could be part of the nervous system and innervated by prominent nerves and muscle fibers, we carried out antibody staining using the widespread neurotransmitter serotonin (5-HT) and labeled the surrounding musculature (F-actin fibers) with phalloidin. Whereas the 5-HT staining revealed a dense meshwork of nerves and somata within the entire pharynx region (Additional file 4: Figure S4), the lobes themselves exhibit only faint 5-HT-IR. Nevertheless, the anti-serotonin staining shows that the lobes are neural innervated, but that prominent somata clusters or neuropils are absent. Phalloidin staining shows dense muscle bundles within the entire pharynx, whereas F-actin labelling is almost lacking within the lobe (Additional file 4: Figure S4).

### Expression levels of GLTx

Transcriptome libraries were used to ascertain the GLTx expression level in putative venom glands (SR21 + SR22), pharyngeal lobes (SR23 + SR24) and body (SR25 + SR26). The highest relative number of mapped reads (212–457 matched reads per million reads) was present in the lobes. Substantially lower numbers (0.47–0.78 matched reads per million reads) were identified in the putative venom glands (Table 1). A comparison of the normalized number of mapped reads (normalized in reference to the total number of filtered reads, Table 1 and Additional file 9) between putative venom glands and pharyngeal lobes indicates that the GLTx expression level, based on the full-length transcript (SR23, contig 5772), is around 960 times higher in the lobes. In contrast, the expression of GLTx transcripts is only around 300 times higher in the lobes than in the putative venom glands for the other two GLTx full-length transcripts (SR23, contig 4317 and contig 4318). Since the body samples yielded no GLTx transcripts (maximal 5 reads) in transcriptome libraries and GLTx clones (Additional file 9), this tissue was used as calibrator sample in quantitative real-time PCR (qPCR) experiments.

**Table 1:** Comparison of GLTx expression in different tissues (pharyngeal lobes versus pvg, putative venom glands) of *G. tridactyla* based on RNAseq data. Filtered Illumina reads were mapped against three GLTx full-length transcripts (SR23, contig 5772, contig 4317, and contig 4318) and three full-length GLTx clones (Cons_clone_5A, 6A, and 7). Note that the Fold Change for each contig equals the relative number of mapped reads from the pharyngeal lobes library divided by the relative number of mapped reads from the library of the putative venom glands (see also Additional file 9)

		Lobes	PVG	Lobes vs. PVG
Contig	Length [bp]	Mapped Reads^a^	Mapped Reads^a^	Fold Change, normalized
Cons_clone_5A	3605	454.87	0.78	579.72
Cons_clone_6A	3598	453.01	0.78	577.35
Cons_clone_7	3607	444.99	0.78	567.12
SR23_contig_5772	3846	456.91	0.47	963.83
SR23_contig_4317	3841	212.89	0.70	302.87
SR23_contig_4318	3841	212.32	0.70	302.05

^a^number of mapped reads per million reads of the respective RNAseq library

**Fig. 6:**
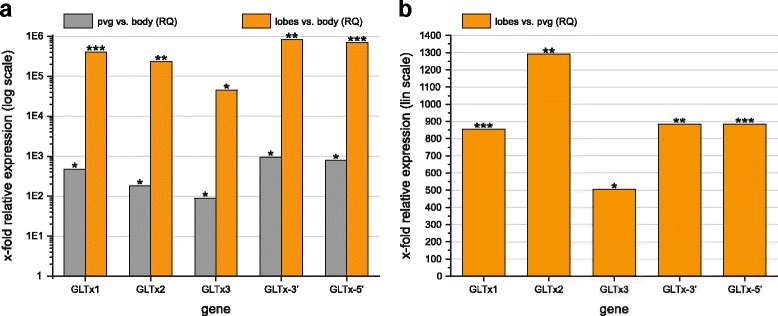
Quantitative real-time PCR (qPCR) expression levels (shown as Fold Change, RQ) between biological groups (putative venom glands (pvg), pharyngeal lobes, and posterior body wall) of five analyzed GLTx transcripts (GLTx paralog 1–3, and adjacent gene regions GLTx-3’ and GLTx-5’; for details see Material and methods section “Quantitative real-time PCR”) in *G. tridactyla* (*n* = 10; ***p ≤ 0.001; **p ≤ 0.01; *p ≤ 0.05). **a** Relative GLTx expression (logarithmic scale) in putative venom glands (grey) and pharyngeal lobes (orange) in comparison to the GLTx expression signal exhibited by the body tissue (RQ = 1). Relative GLTx expression in the pharyngeal lobes and putative venom glands is significantly different from the expression signal in the body tissue. **b** Relative GLTx expression (linear scale) within the pharyngeal lobes in comparison to the putative venom glands (RQ = 1). Relative GLTx expression is significantly different between both putative venom glands and pharyngeal lobes

Analyzing 10 specimens as biological samples with paralog-unspecific GLTx primers spanning an intron-exon-border (GLTx-3’ and GLTx-5’) revealed a higher GLTx expression in both the lobes and the putative venom glands, compared to the body samples, with the highest expression occurring in the lobes (Fig. 6a). A more detailed pattern was found in qPCR experiments using paralog-specific GLTx primers. We recovered GLTx paralog 1 as the most highly expressed paralog, followed by GLTx paralog 2 and paralog 3 in putative venom glands and lobes in comparison to the body samples (Fig. 6a). GLTx expression levels in lobes and putative venom glands are significantly different from the body, as well as from each other (Fig. 6 and Additional file 10b,c). Consistent with our RNAseq results (SR23, contig 5772 and GLTx clones, Table 1), a direct comparison between lobes and putative venom glands (using the latter as calibrator sample) shows that the relative expression level of GLTx is around 500–1300 times higher in the lobes (Fig. 6b). Furthermore, GLTx expression within the pharyngeal lobes and putative venom glands seems to be coordinated, as the most highly expressed paralog in the lobes was also the most highly expressed paralog in the putative venom glands (Additional file 4: Figure S5 and Additional file 10d,e).

## Discussion

### GLTx is an unusual neurotoxin with novel functional organization

In this study we revealed the full coding sequence of glycerotoxin for the first time. Three computationally recovered GLTx full-length transcripts each harbor a signal peptide at the 5’-end (Fig. 1a), which represents a typical feature for secreted toxins [1]. The identification of several paralogs (Fig. 1b) further indicates the existence of a GLTx multigene family, which is another common feature of toxin genes [1]. GLTx constitutes an uncharacterized protein family with 80% of the GLTx gene displaying an unknown domain organization. However, sequence similarity observed in an uncharacterized protein in *Branchiostoma*, a collectin-12 sequence in Cnidaria, and two annelid ESTs outside Glyceridae suggest that the GLTx family may have evolved from an existing gene family. Indeed, such a scenario has been proposed to be a general model for the evolution of venom toxins [1, 31], where gene duplication creates novel paralogs that can evolve new functions via sub- or neo-functionalization. However, further phylogenetic analyses and broader sampling of GLTx within Glyceridae is necessary to confirm this hypothesis, and to elucidate the details of the evolutionary origin of GLTx. Moreover, the GLTx gene shows an intron-exon-structure (Additional file 4: Figure S2), which makes it extremely unlikely that this neurotoxin is encoded and expressed by symbiotic bacteria. Our mass spectrometry results further suggest that the molecular weight of the complete GLTx polypeptide chain (around 150 kDa, Additional file 4: Figure S1) is around half the weight previously reported in the literature (300–320 kDa) [13, 22, 28]. Crucially, only the large molecular weight form is active [13], suggesting that GLTx may function as a dimer. Interestingly, the GLTx polypeptide chain has a similar size as the monomer of α-latrotoxin (LTX), the vertebrate-specific pore-forming neurotoxin isolated from the black widow spider venom (genus *Latrodectus*). LTX is also an excitatory neurotoxin that increases neurotransmitter release [32]. Unlike GLTx, the effects of LTX are irreversible and cause the complete depletion of pre-existent synaptic vesicles [32–34]. Another excitatory neurotoxin is trachynilysin isolated from the venom of the stonefish *Synanceia trachynis*. This neurotoxin may as well form pores by insertion into the cell membrane, and leads to selective depletion of synaptic vesicles in an irreversible manner [35–37].

A unique feature of GLTx is its ability to reversibly up-regulate the activity of presynaptic Ca_v_2.2 channels [13]. Although a detailed structural functional characterization of the glycoprotein and its mode of action are not yet available, the GLTx sequence data presented in this manuscript (Additional files 2 and 3) enable us to speculate about putative mechanisms. The calcium-binding EGF domain of GLTx (Fig. 1a) may allow the binding of the neurotoxin to extracellular recognition sites on the presynaptic plasma membrane as EGF-like domains are supposed to favor protein-protein interactions [38]. The requirement of Ca^2+^ for GLTx activity is in agreement with the observation that GLTx functions in a calcium-dependent manner [26]. The GLTx transcript further encodes a CCP domain (complement control protein, Fig. 1a), also known as sushi domain or short consensus repeat (SCR). CCP modules are involved in specific protein–protein and protein–carbohydrate interactions and are found within regulators of complement activation (RCA) [39, 40]. One of the homologous protein classes that belong to the RCA family is factor H [41], which is a glycoprotein regulator entirely composed of CCP domains [42]. Through its ability to recognize polyanionic markers on host cell surfaces, factor H can interact with host cell membranes or self-surfaces [42]. In venoms, CCP containing proteins have been reported from the venom cocktail of parasitic wasps of the genus *Leptopilina* [43, 44], but functional assays are to our knowledge not yet available. The GLTx transcripts further encode two WSC domains (cell wall integrity and stress response component, Fig. 1a), which represent putative carbohydrate binding domains. The human plasma membrane protein polycystin 1, which also contains a WSC domain, is suggested to function as a mechanosensor regulating proliferation, adhesion and differentiation [45, 46]. In yeast, this domain is found in regulators of cell wall integrity and the stress response pathway [47, 48]. A fungal β-1,3-exoglucanase that hydrolyzes laminarin to glucose monomers also contains tandem WSC domains, like GLTx [49]. The sequence information presented here constitutes an important step towards understanding the GLTx mode of action and illuminates its value as a neurobiological tool.

### The glycerid venom apparatus is a complex system

Our study substantially changes our current understanding of the glycerid venom system. For over a century the glycerid venom apparatus was assumed to be constructed of four putative venom glands that are each connected through a duct to a tooth [14–20]. However, our *in situ* and fluorescence *in situ* hybridization experiments revealed a clear GLTx expression signal restricted to four pharyngeal lobes and an area at the base of the teeth (Fig. 2 and Fig. 4b). Ehlers [15] (using the name “Lappen”) and Gravier [50] (using the name “membrane quadrilobée”) thought these lobate structures were part of the nervous system, a view that is not supported by our immunohistochemical studies (Additional file 4: Figure S4). Oppenheimer [51] already doubted that the lobes are exclusively part of the nervous system as she already distinguished different cell types within the lobe. Raphaël [52] denied that the lobes were part of the nervous system and rather proposed that the lobes function in the fixation and excretion of hemoglobin. Later studies of Michel [20, 53] (using the name “languettes”) again accepted that the basal parts of the pharyngeal lobes are part of the nervous system, and suggested further the presence of glandular cells thought to secrete a proteolytic enzyme having a digestive function. However, the four pharyngeal lobes have never been recognized to be part of the glycerid venom system.

Whereas the pharyngeal lobes show an obvious staining signal, we were not able to detect any GLTx *in situ* signal inside the putative venom glands (Fig. 2). Moreover, comparative transcriptomics and qPCR experiments carried out on three tissue types of *G. tridactyla*, namely putative venom glands, pharyngeal lobes and posterior body wall, show that the relative GLTx expression is 500–1,300 times higher in the lobes than in the putative venom glands (Fig. 6b and Table 1). Furthermore, antibody staining against GLTx highlighted a network of ducts that connects lobes and teeth (Fig. 3c,d and Fig. 5 and Additional file 8). Taken together, these results strongly support the conclusion that the lobes are the main site for neurotoxin expression, and that the neurotoxin might be transferred from the pharyngeal lobes directly to the teeth where it is injected into prey through a series of pores [14, 17, 23]. Our milking of glycerid venom confirms that there is a direct link between putative venom glands and teeth, a connection that may be independent of the link between pharyngeal lobes and teeth. A network of canals between putative venom glands and pharyngeal lobes could not be identified even though both tissue types show a correlated GLTx expression profile (Additional file 4: Figure S5). This is especially interesting as GLTx antibody staining on paraffin embedded pharynx clearly recovered the neurotoxin inside the lumen of the putative venom glands (Additional file 4: Figure S3). Yet, the GLTx protein detected in the lumen of the putative venom glands may have been produced in the pharyngeal lobes, as there was no GLTx *in situ* signal detectable inside the putative venom glands (Fig. 2). Furthermore, our investigation cannot exclude the possibility that the low GLTx expression signal inside the putative venom glands (qPCR studies and transcriptome analyses, Fig. 6 and Table 1) comes from the ducts connecting the lobes and teeth. However, a recent transcriptome study on venom gland tissue of *G. tridactyla* revealed a complex mixture of putative toxin transcripts [54], which suggests a glandular function of the putative glands alongside its function as a venom storage site. Whether or not the pharyngeal lobes and putative venom glands may be involved in the differential expression of different venom toxins needs further investigation. Our results clearly show that the functional morphology of the glycerid venom system is more complex than hitherto thought.

Compartmentalization of toxin production has been reported from different venomous taxa [55–57]. Within the large protostome clade Lophotrochozoa, complex venom systems comprising several structural subunits have also been described. The cephalopod venom apparatus comprises two pairs of histologically different venom glands, named posterior and anterior venom glands [58]. Whereas analyses on the posterior venom glands in cephalopods revealed toxins that convergently evolved in other venomous animals [59–61], the role of the anterior glands remains poorly investigated. These glands are considered as mucus secreting organs [58] but their contribution to the cephalopod venom cocktail remains unclear, even though it was recently shown that they may also express some toxin transcripts [62].

In Glyceridae, we show distinct GLTx expression patterns in pharyngeal lobes and putative venom glands (Fig. 6 and Table 1). In this respect, the glycerid venom apparatus resembles to a degree the venom system of carnivorous cone snails. Cone snails are able to produce two types of venom in distinct parts of the venom duct [63]. A defensive toxin cocktail containing paralytic peptides and neurotoxins from the proximal part of the duct, and a less complex predatory venom cocktail from the distal part of the duct [55]. The cone snails are able to secrete these venom types selectively depending on whether predatory or defensive stimuli are received. Since the GLTx expressing lobes are innervated by the nervous system (Additional file 4: Figure S4), it is possible that bloodworms are capable of a rapid stimuli-evoked (defensive or predatory) secretion of GLTx. It is also possible that neurotoxin-rich secretions are only selectively added to the venom mix synthesized and stored in the putative venom glands. Concordantly, *Glycera alba* is supposed to deplete venom glands incompletely during the first bite which evokes discoordination rather than death and paralysis of the prey, whereas quantitatively more venom seems to be delivered after a firm grip [64]. The remarkable variability in GLTx expression levels revealed by the qPCR experiments of 10 studied specimens (Additional file 4: Figure S5) may indicate that these specimens are in distinct phases of venom replenishment. These results highlight the possibility that Glyceridae are able to meter their venom stocks.

Further research is necessary to test if the neurotoxin GLTx is unique in being differentially expressed in glycerid putative venom glands and pharyngeal lobes, or whether the expression of other toxins also corresponds to this anatomical differentiation of the venom apparatus. To address this question, comparative transcriptomic and proteomic analyses of other venom toxins as well as detailed histological studies of the pharyngeal structures are required. The recent transcriptomic study of von Reumont et al. [54] focused exclusively on glycerid venom gland tissue, hence their results need to be reassessed in view of the role of the pharyngeal lobes in the synthesis of venom toxins reported here. Our current results clearly demonstrate that multidisciplinary analyses are invaluable for understanding the glycerid venom apparatus in particular, and venom systems in general.

## Conclusions

In this work, we report the full sequence of glycerotoxin (GLTx), a neurotoxin known to act specifically as a Ca_v_2.2 agonist. GLTx represents a toxin family comprising at least three different paralogs with uncertain evolutionary origin. Moreover, our data show that GLTx likely functions as a dimer with the subunits being held together by non-covalent bonds. GLTx transcripts are expressed in two locations in the glycerid venom apparatus, the putative venom glands and pharyngeal lobes, a previously unrecognized component of the venom system. GLTx protein is restricted to the pharyngeal lobes and to the lumen of the putative venom glands. Furthermore, GLTx is expressed 500 to 1,300 times higher in the pharyngeal lobes than in the putative venom glands. Our results overturn more than a century of textbook consensus, suggesting that a fundamental revision of our understanding on the functional organization of the venom system in bloodworms is urgently needed.

## Methods

### Protein studies

#### Protein sequencing and characterization

For its initial characterization, GLTx was purified on an 8% SDS-PAGE, silver stained [13], cut out and after destaining, in gel digested with trypsin. Samples were analyzed by nano-LC MS/MS using a quadrupole time-of-flight mass spectrometer (Micromass) and *de novo* sequencing visually inspected by the Cancer Research UK London Research Institute mass spec facility.

Lyophilized venom of *Glycera tridactyla* Schmarda, 1861 (Annelida, Glyceridae) was further dissolved in ultrapure water to a concentration of 5 mg/ml, and 50 μg separated by SDS-PAGE using a 12.5% Tris-glycine gel under reducing conditions. Bands were visualized by staining with colloidal Coomassie followed by destaining of the gel by 1% (*vol/vol*) acetic acid. Individual bands were dissected, digested with trypsin, and tryptic peptides eluted as described previously [65]. Proteins were identified by analyzing the tryptic peptides by LC-ESI-MS/MS and matching the resulting fragment spectra with sequences obtained by translated tissue transcriptomes (see below). LC-MS/MS experiments were carried out on an AB Sciex 5600 TripleTOF equipped with a nano-source heated to 150 °C. Venom was fractionated on a Shimadzu Prominence nano-HPLC with a 1 μm internal diameter 100 mm Agilent 3 μm 90 Å C18 reverse phase column at a flow of 500 nl/min and a gradient of 2-40% solvent B (0.1% formic acid (FA), 90% acetonitrile) in 0.1% FA over 10 min. MS1 scans were acquired at 350–1800 m/z with an accumulation time of 250 ms. MS2 scans were acquired on up to 20 ions per cycle that were of 80–1400 m/z with 2–5 charges and intensity greater than 120 counts per second, accumulating ions for 100 ms. Spectra were searched against a pooled-tissue transcriptomic sequence database with ProteinPilot v.5.0 (AB Sciex, Mt Waverley, Victoria, AUS) using thorough search settings and allowing for biological modifications. Decoy-based false discovery rates (FDR) were estimated by ProteinPilot, and only protein identifications ranked above the 1% local FDR threshold were considered.

### Transcriptome sequencing and GLTx characterization

#### Specimen collection and tissue preparation

Specimens of *Glycera tridactyla* Schmarda, 1861 (Annelida, Glyceridae) were obtained from the Roscoff marine biological station (Station Biologique Roscoff, France) in February 2015. To minimize influences of stress, the animals were maximally kept for 6 days in seawater aquaria. The small-sized pharyngeal lobes (four per specimen) were left attached to the inner pharynx epithelium to ensure their complete removal during dissection. The putative venom glands (four per specimen) were cut below the basis of the teeth to exclude ducts connecting the pharyngeal lobes and teeth (Additional file 4: Figure S6). As reference tissue, a part of the posterior body wall was dissected and the gut and parapodia were removed (Additional file 4: Figure S6). Dissected tissues were immediately homogenized in TRIzol® LS Reagent (Life Technologies, Darmstadt, Germany), and stored at −20 °C before proceeding to RNA isolation.

#### RNA extraction, library reconstruction and Illumina sequencing

Total RNA was extracted from the pharyngeal lobes, the putative venom glands, and a posterior part of the body wall (Additional file 4: Figure S6) using TRIzol® LS Reagent (Life Technologies, Darmstadt, Germany). To remove genomic DNA residues from the samples, a DNA digestion step using DNase I (Roche, Mannheim, Germany) was carried out in a RNase-free environment before purification with the RNeasy MinElute Cleanup Kit (Qiagen, Hilden, Germany) according to the manufacturer’s protocol. RNA concentration and quality were determined on a NanoDrop 2000 (Thermo Scientific, Wilmington, DE) and an Agilent 2100 Bioanalyzer (High Sensitivity RNA Chip, Agilent Technologies, Santa Clara, CA).

Transcriptome libraries were constructed for a single specimen (SR21, putative venom glands; SR23, pharyngeal lobes; and SR25, posterior body wall), and for pooled samples comprising RNA of three individuals (SR22, putative venom glands; SR24, pharyngeal lobes; and SR26, posterior body wall). For purification of mRNA out of total RNA, the Dynabeads® mRNA Purification Kit (Invitrogen, Carlsbad, CA) was used for higher concentrated samples and Sera-Mag Oligo(dT) Beads (Distrilab, Leusden, Netherlands) were used for lower concentrated samples (putative venom glands, SR21 and SR22). First strand cDNA synthesis reactions, implementing an 8 min (at 85 °C) fragmentation step, were performed with random hexamer primers (Thermo Fisher Scientific, Wilmington, DE) and SuperScript® III reverse transcriptase (Invitrogen, Carlsbad, CA). Subsequently, second strand cDNA synthesis reactions were performed with DNA polymerase I and ribonuclease H (Life Technologies, Carlsbad, CA), and reaction products were purified with the QIAquick® PCR Purification Kit (Qiagen). Starting from the blunt-end repair, Illumina libraries were processed according to the Illumina multiplex protocol of Meyer, Kircher [66] using double indexed library adapters [67]. Libraries were sequenced together on one lane of the HiSeq 2500 (Illumina, San Diego, CA) at the Max Planck Institute for Evolutionary Anthropology (Leipzig, Germany). Afterwards, Illumina paired-end reads (140 bp) were sorted according their indices, adapters were clipped and base calling was conducted with freeIbis [68]. Reads with false paired indices were discarded, and overlapping paired-end reads were trimmed and merged to a single sequence [69].

#### Processing of sequencing data

Illumina raw reads were trimmed (10 bp) at both ends and single sequences shorter than 60 bp were removed using cutadapt v.1.8.1 [70], respectively. Afterwards, Illumina sequences were filtered with ConDeTri v.2.2 [71] and only reads of which 95% of the nucleotides have a PHRED score [72, 73] above 15 were kept for further analyses (Additional file 11). The processed (using cutadapt v.1.8.1 and ConDeTri v.2.2) Illumina reads were assembled *de novo* using IDBA-tran v.1.1.1 [74]. IDBA-tran assemblies are constructed using an initial k-mer size of 20, an iteration size of 5, and a maximum k-mer size of 120 (Additional file 11).

#### Identification of GLTx in transcriptome libraries and cDNA

Assemblies were screened for putative GLTx transcripts through BLAST-searches (tblastn) v.2.2.28+ [75] using short amino acid sequences of the GLTx protein (see Material and methods section “Protein sequencing” and Additional file 1) as reference.

A nearly full-length GLTx transcript (around 3600 bp; using SR23, contig 5772 as reference) was amplified in second strand cDNA (primer pair full-transF/full-transR; Additional file 12) prior to cloning (for details see Material and methods section “Morphological analyses, cloning”). Finally, three GLTx clones (Cons_clone_5A, 6A, and 7) were analyzed through primer walking (Additional file 12). Amplicons were sequenced at the GATC Biotech AG (Constance, Germany).

#### Annotation of GLTx full-length transcripts

GLTx full-length transcripts (SR23, contig 5772, contig 4317, and contig 4318) and three nearly full-length GLTx clones (Cons_clone_5A, 6A, and 7; Additional file 3) were annotated using common online tools. Signal peptides were identified through the SignalP v.4.1 server [76]. Identification of signaling domains was carried out using Pfam v.30.0 database [77] and SMART, the simple modular architecture research tool [78, 79]. Similarity to published sequence data was analyzed through BLAST-searches (blastp) v.2.6.0+ in NCBI GenBank. Molecular weight was calculated based on the full-length transcripts (without signal peptide) using CLC Main Workbench v.7.7 (CLCbio, Qiagen, Aarhus, Denmark; www.clcbio.com).

#### GLTx paralog screening

To screen for putative GLTx paralogs, a Maximum likelihood analyses v.8.2.8 [80] of different clones was performed (raxmlHPC-PTHREADS-AVX, GTR + GAMMA + I, 1,000 pseudoreplicates). The phylogenetic analysis comprises the same 746 bp GLTx gene fragment (Additional file 6) analyzed in 49 GLTx clones (primer pair 2 F/4R; see Material and methods section “Morphological analyses, cloning”), three GLTx clones (primer pair full-transF/full-transR; see Material and methods section “Identification of GLTx in cDNA”), and three GLTx full-length transcripts (SR23, contig 5772, contig 4317, and contig 4318; see Material and methods section “Identification of GLTx in transcriptome libraries”). The unrooted paralog tree was visualized and edited with iTOL v.3.2.4 [81–83].

#### Genomic structure of the GLTx gene

The genomic structure of the GLTx gene was analyzed in genomic DNA of *G. tridactyla*, collected in April 2011 nearby the Roscoff marine station. DNA was extracted using the NucleoSpin® Tissue Kit (Macherey-Nagel, Düren, Germany) according to the manufacturer’s protocols. Based on transcriptome sequence information, primers were designed using NetPrimer (PREMIER Biosoft, Palo Alto CA; Additional file 12), and PCR experiments carried out. Unknown gene parts that are adjacent to known gene regions were determined using the GenomeWalker™ Universal Kit (Clontech Laboratories, Inc., Takara Bio Company, Mountain View, CA) [84]. Amplicons were purified using the NucleoSpin® Gel and PCR Clean-up kit (Macherey-Nagel) according to the manufacturer’s protocols. Sanger sequencing was performed by the GATC Biotech AG (Constance, Germany).

### Morphological analyses on the glycerid venom apparatus

#### Specimen collection and fixation

For *in situ* hybridization experiments and antibody staining, adult specimens of *Glycera tridactyla* Schmarda, 1861 (Annelida, Glyceridae) were collected intertidally from muddy areas of the rocky shore nearby Roscoff marine biological station in spring 2013. For fluorescence *in situ* hybridization (FISH) experiments and antibody staining, adult individuals of *G. tridactyla* were collected from sandy beach sections of the intertidal zone nearby the Wimereux marine biological station (Station Marine de Wimereux, Université de Lille, France) in March 2014.

Specimens used for *in situ* and FISH were fixed in 4% paraformaldehyde (PFA) in 0.1 M phosphate buffered saline (PBS) for 4 h at 4 °C, washed 5 min in a solution equally proportioned PBS and methanol, before transferred in 100% methanol, and stored at −20 °C. Samples used for antibody staining were fixed in 4% PFA in 0.1 M PBS overnight at 4 °C, washed 3 times for at least 2 h in PBS at 4 °C, and stored in PBS containing 0.005% sodium azide (NaN_3_) at 4 °C.

#### RNA isolation, amplification of a GLTx gene fragment, cloning and probe construction

Total RNA was extracted from whole pharynx tissue of an adult *G. tridactyla* using TRIzol® Reagent (Invitrogen, Carlsbad, CA), and purified through the RNeasy MinElute Cleanup Kit (Qiagen) according to the manufacturer’s protocols. The specimen was collected nearby the Roscoff marine biological station in December 2010, and fixed in RNAlater (Ambion, Darmstadt, Germany). First strand cDNA synthesis was performed using random hexamer primers (Fermentas, St. Leon-Rot, Germany) and SuperScript^TM^ III reverse transcriptase (Invitrogen, Carlsbad, CA). Second strand synthesis was carried out with ribonuclease H (Invitrogen, Carlsbad, CA) and DNA polymerase I (Invitrogen, Carlsbad, CA), and the second strand cDNA product subsequently purified through the NucleoSpin® Gel and PCR Clean-up kit (Macherey-Nagel) according to the manufacturer’s protocols.

A 746 bp exonic gene fragment of GLTx identified in *G. tridactyla* (exon 3, Additional file 4: Figure S2) was amplified through the primer pair 2 F/4R (Additional file 12) in second strand cDNA and genomic DNA, and subsequently purified through the NucleoSpin® Gel and PCR Clean-up kit (Macherey-Nagel). The purified PCR products were cloned into the pGEM® -T Vector (pGEM®-T Vector System I, Promega Corporation, Madison, WI) and transformed in *E. coli* JM109. Finally, 49 clones were transferred in HPLC-H_2_O, frozen (−20 °C), defrost, amplified through the M13 primer pair (M13F/M13R), and sequenced at the GATC Biotech AG (Constance, Germany). Two clones were used for preparation of digoxigenin-labeled RNA probes through the DIG RNA Labeling Kit SP6/T7 (Roche) according to the manufacturer’s protocols.

#### In situ hybridization

Expression studies were performed on pharynx tissues of *G. tridactyla*. Per specimen, the pharynx was dissected into two halves.

The pharynx samples were rehydrated stepwise by washing at room temperature for 5 min in mixtures of a serial dilution (see Additional file 13: Protocol S1) of methanol and PTW (1 × PBS + 0.1% Tween-20), followed by 4 × washing in 100% PTW. Samples were digested with proteinase K (0.01 mg/ml in PTW) for 5 min without shaking, and stopped by washing twice for 5 min in glycine/PTW (2 mg/ml). The pharynx samples were then washed in 1% triethanolamine in PTW, and glacial acetic acid was added twice with an incubation time of 5 min each to permeabilize the cells. Samples were washed twice in 100% PTW for 5 min, and then re-fixated through 60 min incubation in 4% PFA in PTW, at room temperature on a shaker. Afterwards, the pharynx samples were again washed 5 × 5 min in PTW, transferred in new 2 ml non-sticky tubes filled with PTW, incubated for 5 min on a shaker before heated to 80 °C for 10 min without shaking. After removing liquids, the samples were incubated in hybridization buffer for 10 min at room temperature. Liquids were discarded, 65 °C pre-warmed hybridization buffer added, and pre-hybridization was carried out overnight at 65 °C. For hybridization, digoxigenin-labeled RNA probes (SP6/T7, concentration: 1 ng/μl in hybridization buffer) were denaturated by heating for 10 min at 80 °C without shaking. Per analyzed specimen, one pharynx half was transferred in SP6 probe, the other in T7 probe (sense and antisense), and hybridization was performed for 72 h at 65 °C. After removing probes, the pharynx tissues were washed at 65 °C for 5 min and 20 min in 65 °C pre-warmed hybridization buffer. The pharynx samples were then washed at 65 °C stepwise in a serial dilution of 65 °C pre-warmed mixtures of hybridization buffer and 2 x SSC (sodium saline citrate) for 10 min, followed by washing 10 min in 65 °C pre-warmed 100% 2 × SSC, and 2 × 30 min in 65 °C pre-warmed 0.02 × SSC. At room temperature, additional 5-min wash steps in mixtures of a serial dilution of 0.02 × SSC and PTW were carried out, followed by washing 6 × 5 min in 100% PTW. For visualization, the samples were blocked for 1 h at room temperature on a shaker in blocking buffer (5% normal goat serum in PTW). The Anti-Digoxigenin-AP Fab fragments antibody (Roche, Mannheim, Germany) diluted at 1:5,000 in blocking buffer was added, and incubation was carried out overnight at 4 °C on a shaker. After washing 8 × 10 min in PTW at room temperature, followed by washing 3 × 5 min in AP staining buffer, color staining was initiated by adding the NBT/BCIP staining solution (Carl Roth, Karlsruhe, Germany). Light-sensitive staining reaction was kept in the dark without shaking, and stopped after 45 min–3 h through 4% PFA in PTW. After 60 min incubation, the pharynx samples were washed once in PTW, placed overnight at 4 °C on a shaker, and washed again 2 × 2 h in PTW at 4 °C. Until imaging, the pharynx samples were stored in the dark at 4 °C without shaking. After imaging, the pharynx tissues were transferred in 0.1 M PBS containing 0.005% NaN_3_, and stored in the dark at 4 °C.

To detect signal, the pharynx samples were analyzed under a stereomicroscope (Leica WILD M10, Leica Microsystems, Wetzlar, Germany) equipped with a color digital camera (SensiCam, 12 bit cooled imaging, PCO AG, Kelheim, Germany), and the CamWare v.3.11 software. Final panels were designed with Adobe Photoshop CS5.1 and Adobe Illustrator CS5.1.

#### Anti-GLTx staining

Antibody staining (see Additional file 13: Protocol S2) was carried out on pharynx tissues of *G. tridactyla*. The everted pharynx was dissected into two equally sized halves.

For tissue permeabilization, pharynx samples were incubated overnight at room temperature in a solution of 0.1 M PBS containing 0.1% Triton X‐100 (PTA), 0.1% NaN_3_, and 6% normal goat serum (Sigma‐Aldrich, St. Louis, MO, USA) (block‐PTA). The primary antibody monoclonal mouse anti‐GLTx (4G9, [13]; diluted 1:500), was applied for 48–72 h at 4 °C on a shaker. Samples were then washed 3 × 2 h at room temperature in 0.1 M PBS containing 0.1% Triton X‐100 (PTA), and block‐PTA. The secondary fluorochrome conjugated antibody (goat anti‐mouse Alexa Fluor 488; Invitrogen, Carlsbad, CA; diluted 1:500) was added and incubation was carried out in the dark for 72 h at 4 °C on a shaker. Subsequently, tissue samples were washed 2 × 1.5 h–2 h in 0.1 M PBS, followed by 2-h incubation in phalloidin–rhodamine (5 μl phalloidin stock solution per 500 μl PBS; Invitrogen, Darmstadt, Germany) for additional staining of muscle tissue. At last, tissue samples were dehydrated in an ascending series of isopropanol, treated for 10 min in Murray’s clearing solution (benzyl alcohol + benzyl benzoate, at a ratio of 1:2), and finally mounted between two coverslips in dibutyl phthalate xylene (DPX; Sigma-Aldrich).

Specimens were analyzed with the confocal laser scanning microscope Leica TCS STED (Leica Microsystems, Wetzlar, Germany), and confocal image stacks were processed with Leica AS AF v.2.3.5 (Leica Microsystems) and Imaris v.6.3.1 (Bitplane AG, Zurich, Switzerland). Final panels were designed with Adobe Photoshop CS5.1 and Adobe Illustrator CS5.1.

#### Antibody staining (II) against GLTx

Antibody staining was further performed on sections of putative venom glands of *G. tridactyla* [13], following classical paraffin-embedded tissue sections, treated with blocking buffer (0.1 M PBS, 3% normal goat serum, and 0.1% Triton X‐100). The primary antibody monoclonal mouse anti‐GLTx (4G9, see Material and methods section “Anti-GLTx staining”) was applied overnight at 4 °C, washed several times with blocking buffer by addition of the secondary antibody (goat anti‐mouse Alexa Fluor 488; Invitrogen, Carlsbad, CA). Sections were examined with a Zeiss LSM 510 confocal microscope (Carl Zeiss, Jena, Germany).

#### Antibody staining against serotonin

Antibody staining against serotonin was performed on bisected pharynges of *G. tridactyla* as described in the Material and methods section “Anti-GLTx staining”, using the primary antibody polyclonal rabbit anti-serotonin (INCSTAR, Stillwater, USA; diluted 1:500), and the secondary fluorochrome conjugated antibody goat anti‐rabbit Alexa Fluor 633 (Invitrogen, Carlsbad, CA; diluted 1:500). The samples were embedded, analyzed and processed as described in the Material and methods section “Anti-GLTx staining”.

#### Fluorescence *in situ* hybridization (FISH) coupled with antibody staining against GLTx

Double-staining (see Additional file 13: Protocol S3) was performed on an entire pharynx of *G. tridactyla*.

The first part of protocol is concordant with the *in situ* hybridization protocol (Additional file 13: Protocol S1), with modifications regarding the blocking reagent, the anti-digoxigenin antibody, and color staining solution. Before blocking, the pharynx sample was washed five times for 5 min in TNT, instead of PTW. Furthermore, blocking was carried out for 3 h in TNB blocking buffer (0.5% blocking reagent, PerkinElmer, Beaconsfield, MA), and incubated overnight with the Anti-Digoxigenin-POD, Fab fragments antibody (Roche, Mannheim, Germany), diluted at 1:100 in TNB blocking buffer. Other than in the *in situ* protocol, color staining was performed in FITC-tyramide staining solution, diluted at 1:50 in 1 × Plus Amplification Diluent (TSA™ Plus Fluorescein System, PerkinElmer), and stopped after 30 min through washing for 5 min, and 10 min in TNT. After color staining, the pharynx sample was kept in the dark. Next, the sample was washed for 5 min at room temperature in mixtures of a serial dilution of TNT/0.1 M PBS, followed by washing 3 × for 5 min in 0.1 M PBS and proceeding with a modified version of the anti-GLTx staining protocol (Additional file 13: Protocol S2). As modification, the tissue was permeabilized for only 30 min, and the primary antibody and the secondary fluorochrome conjugated antibody (goat anti‐mouse Alexa Fluor 568; Invitrogen, Carlsbad, CA; diluted 1:500) were added overnight, instead of 72 h. As a further modification, additional nuclear counterstaining was carried out through 1-h incubation with TO-PRO®-3 Iodide (Life Technologies, Darmstadt, Germany). The sample was analyzed and processed as described at the end of the anti-GLTx staining protocol (see Material and methods section above).

### Expression studies

#### Specimen collection and tissue preparation

Quantitative real-time PCR (qPCR) experiments were performed on *Glycera tridactyla* Schmarda, 1861 (Annelida, Glyceridae) specimens obtained from the Roscoff marine biological station in February 2015. Three different tissue types (pharyngeal lobes, putative venom glands, and posterior body wall) were dissected as described in detail for library preparation (see according Material and methods section “Transcriptome sequencing”).

#### RNA isolation and first strand cDNA synthesis

Total RNA (600 ng each) obtained from pharyngeal lobes, putative venom glands, and a posterior part of the body wall (for details see Material and methods section “Transcriptome sequencing”) was used in first strand cDNA synthesis reactions carried out with random hexamer primers (Thermo Fisher Scientific) and SuperScript® III reverse transcriptase (Invitrogen, Carlsbad, CA). The first strand cDNA synthesis products were used for quantitative real-time PCR (qPCR) experiments.

#### Quantitative real-time PCR with SYBR green

Quantitative real-time PCR (qPCR) studies were performed with Platinum® SYBR® Green qPCR SuperMix-UDG with ROX (Invitrogen, Carlsbad, CA) using half the sample size (25 μl each) as described in the manufacturer’s protocol. Ten biological samples (= specimens) were analyzed with two technical replicates per studied amplicon, each around 210 bp in size. Analyses were performed on the same exonic gene region (primers see Additional file 10) of three GLTx paralogs (see Results section “Characterization of glycerotoxin”) and two adjacent gene regions amplified with one primer spanning an intron-exon-boundary (primers see Additional file 10). Amplification of the normalizer genes (reference genes/endogenous control) — the single copy ribosomal protein genes rps3 and rps15a — was also carried out with an intron-exon spanning primer. Samples were run on the Applied Biosystems 7300 Real-Time PCR System (Applied Biosystems, Darmstadt, Germany) under the following cycling conditions: 1 cycle at 50 °C/2 min, 1 cycle of denaturation at 95 °C/2 min, followed by 40 two-segment cycles of amplification (95 °C/15 s, 60 °C/30 s), and a final dissociation cycle. Baseline and threshold were adjusted automatically and Ct values were determined for each sample using the accompanying 7300 System SDS v.1.4 software (Applied Biosystems). Delta-Ct values (ΔCt) used to normalize gene expression were calculated as follows: ΔCt = average Ct_target gene_ – Normalization Factor (NF), where NF is the mean Ct of both endogenous controls used (rps3 and rps15a) as described by Vandesompele et al. [85]. The average Ct_target gene_ comprises of two technical replicates per gene for each studied specimen. The relative quantification shown as Fold Change (RQ) between biological groups (putative venom glands, pharyngeal lobes, and body tissue from all analyzed specimens) were calculated automatically with the DataAssist™ v.3.01 software (Applied Biosystems) according to the following formula: RQ = geometric mean 2^(−ΔCt)^ / geometric mean 2^(−ΔCt reference)^. Thereby, the replicates of all biological samples of a group contributed to their respective geometric mean calculation. RQ significance was assessed by a two-sample, two-tailed Student’s *t*-test comparing the 2^(−ΔCt)^ values of the groups and p-values were adjusted using Benjamini-Hochberg False Discovery Rate [86] using the DataAssist™ v.3.01 software (Applied Biosystems).

#### Expression level estimates based on transcriptome libraries

GLTx expression was estimated from *G. tridactyla* transcriptome libraries (for details on library preparation see Material and methods section “Transcriptome sequencing”) of three different tissue types (putative venom glands, pharyngeal lobes, and posterior body wall). Before mapping, the processed and filtered Illumina reads of a single specimen library and the referring pooled library were merged (SR21 + SR22, putative venom glands; SR23 + SR24, pharyngeal lobes; SR25 + SR26, posterior body wall; Additional file 11). Merged Illumina reads were mapped against three GLTx full-length transcripts (SR23, contig 5772, contig 4317, and contig 4318) and three nearly full-length GLTx clones (Cons_clone_5A, 6A, and 7). Mapping was carried out using segemehl v.0.2.0 [87, 88] and an identity score of 95% (A = 95). Mapping results were visualized using Tablet v.1.15.09.01 [89]. To estimate the Fold Changes of GLTx transcription between the pharyngeal lobes and putative venom glands, the relative numbers of matched reads per contig from either of these libraries were calculated and thus put in relation to the other (matched reads _lobes_ divided by matched reads _gland_; Table 1 and Additional file 9).

## Additional files

Additional file 1: Position of short amino acid sequences alongside the GLTx full-length transcripts. Short amino acid sequences were recovered through protein sequencing of purified glycerotoxin extracted out of the venom gland cocktail in *G. tridactyla*. Short amino acid sequences varying in length between 7–18 amino acids served as reference in BLAST-searches to identify the neurotoxin in transcriptome libraries of *G. tridactyla*. (TXT 30 kb)
Additional file 2: Nucleotide alignment of GLTx full-length transcripts (SR23, contig 5772, contig 4317, and contig 4318) and three nearly full-length GLTx clones amplified from second strand cDNA (Cons_clone_5A, 6A, and 7) in *G. tridactyla*. (TXT 23 kb)
Additional file 3: Protein-translated alignment of GLTx full-length transcripts (SR23, contig 5772, contig 4317, and contig 4318) and three nearly full-length GLTx clones amplified from second strand cDNA (Cons_clone_5A, 6A, and 7) in *G. tridactyla*. (TXT 7 kb)
Additional file 4: Figure S1. SDS-PAGE of reduced *G. tridactyla* venom. **Figure S2.** Genomic organization of the glycerotoxin gene analyzed in *G. tridactyla*. **Figure S3.** Immunolocalization of GLTx in a cross section through a putative venom gland embedded in paraffin. **Figure S4.** Anti-serotonin (5-HT) staining and phalloidin–rhodamine counterstaining on everted *G. tridactyla* pharynges cut into two halves. **Figure S5.** Quantitative real-time PCR (qPCR) expression levels (shown as Fold Change, RQ) between biological groups (putative venom glands [A], pharyngeal lobes [B], and posterior body wall [C]) per analyzed specimen (*n* = 10, biological samples). **Figure S6.** Tissues analyzed in comparative GLTx expression studies (qPCR experiments and transcriptome analyses) on *G. tridactyla*. (PDF 2931 kb)
Additional file 5: MS-MS best hits of the 150 kDa fractions (**sheet a** and **b**) of *G. tridactyla* venom. Spectra were searched against a pooled-tissue transcriptomic sequence database of the pharyngeal lobes (SR23, single specimen and merged SR23 + SR24, pooled multiple specimen) with ProteinPilot v.5.0. (XLSX 14 kb)
Additional file 6: Alignment comprising a 746 bp gene fragment of 52 GLTx clones and three GLTx full-length transcripts (SR23, contig 5772, contig 4317, and contig 4318). The alignment was used for the maximum likelihood analysis shown in Fig. 1b. (TXT 42 kb)
Additional file 7: Alignment of amplicons generated in genomic DNA of *G. tridactyla*. Sanger sequences revealed an intron-exon-structure for the GLTx gene (see Additional file 4: Figure S2). (TXT 68 kb)
Additional file 9: Comparative GLTx expression analyses carried out on transcriptome libraries of three different tissue types (putative venom glands (pvg), pharyngeal lobes, and posterior body wall) originating from four pooled *G. tridactyla* specimens. Filtered Illumina reads were mapped against three GLTx full-length transcripts (SR23, contig 5772, contig 4317, and contig 4318) and three full-length GLTx clones (Cons_clone_5A, 6A, and 7). Note the remarkably higher number of matched reads in the pharyngeal lobes in comparison to the putative venom glands and body tissue. (XLSX 11 kb)
Additional file 10: Raw data and summary of qPCR experiments performed on three tissue types (putative venom glands [group A], pharyngeal lobes [group B], and posterior body wall [group C]) of *G. tridactyla*. **sheet a** Ct values determined per analyzed gene and technical replicate for 10 biological samples using the 7300 System SDS v.1.4 software (Applied Biosystems). **sheet b** and **c** The Fold Change (RQ, relative quantification) between biological groups (putative venom glands [A], pharyngeal lobes [B], and posterior body wall [C]) and p-values were calculated with the DataAssist™ v.3.01 software (Applied Biosystems). Two technical replicates per analyzed biological sample (n = 10) contribute to the calculations of one group. The single copy ribosomal genes rps3 and rps15a were used as normalizer genes. Software parameters: Maximum allowable Ct value, 40.0; Include max Ct values in calculations, Yes; Exclude outliers among replicates, Yes; Adjust p-values using Benjamini-Hochberg False Discovery Rate, Yes; Normalization method, Endogenous Control; Selected controls, rps15a and rps3. **sheet b** RQ-values and p-values calculated for the putative venom glands [A] and pharyngeal lobes [B] in comparison to the body tissue [C]. **sheet c** RQ-values and p-values calculated for the pharyngeal lobes [B] in comparison to the putative venom glands [A]. **sheet d** and **e** The Fold Change (RQ, relative quantification) between biological groups (putative venom glands [A], pharyngeal lobes [B], and posterior body wall [C]) per analyzed specimen (n = 10, biological samples). Two technical replicates were performed per analyzed specimen. The single copy ribosomal genes rps3 and rps15a were used as normalizer genes. **sheet f** Primer sets used for qPCR amplification of five GLTx specific gene fragments and two normalizer genes (rps3, rps15a). (XLSX 63 kb)
Additional file 11:
**sheet a** Summary statistics of filtering steps (using cutadapt v.1.8.1 and ConDeTri v.2.2) performed on raw Illumina sequencing data of three different tissue types (putative venom glands, pharyngeal lobes, and posterior body wall) analyzed in *G. tridactyla*. Transcriptome libraries were constructed for a single individual (SR21, putative venom glands; SR23, pharyngeal lobes; and SR25, posterior body wall), and based on pooled samples comprising RNA of three individuals (SR22, putative venom glands; SR24, pharyngeal lobes; and SR26, posterior body wall). Abbreviations: PE, paired-end reads; SR, single reads. **sheet b** Summary statistics of IDBA-tran v.1.1.1 assemblies performed on processed Illumina sequencing data originating from *G. tridactyla*. Assemblies were done for three different tissue types of a single individual (SR21, putative venom glands; SR23, pharyngeal lobes; and SR25, posterior body wall), and for Illumina sequencing data of three pooled individuals (SR22, putative venom glands; SR24, pharyngeal lobes; and SR26, posterior body wall). In addition, the filtered reads of single and pooled libraries were merged (SR21 + SR22, putative venom glands; SR23 + SR24, pharyngeal lobes; SR25 + SR26, posterior body wall) to perform comparative expression studies by mapping them against GLTx reference sequences. (XLSX 17 kb)
Additional file 12: Set of primers used to analyze the molecular organization of the GLTx gene. **sheet a** Primer used to amplify GLTx fragments before cloning into the pGEM® -T Vector (pGEM®-T Vector System I, Promega Corporation, Madison, WI). **sheet b** Primer used for primer walking (Cons_clone_5A, 6A, and 7). **sheet c** Primer used to analyze the intron-exon-structure in genomic DNA of *G. tridactyla*. GW, Genome walking. (XLSX 14 kb)
Additional file 13:
**Protocol S1–S3.** Protocol S1. Protocol: *In situ* hybridization on pharynx tissue of *G. tridactyla* (Glyceridae, Annelida). Protocol S2. Protocol: Anti-GLTx staining on pharynx tissue of *G. tridactyla* (Glyceridae, Annelida). Protocol S3. Protocol: Fluorescence *in situ* hybridization (FISH) coupled with antibody staining against GLTx on pharynx tissue of *G. tridactyla*. (PDF 590 kb)
