# Supplementary material for: Comparative analyses of glycerotoxin expression unveil a novel structural organization of the bloodworm venom system
Source: BMC Evol Biol. 2017 Mar 4;17:64. doi: 10.1186/s12862-017-0904-4 (PMC5336659; doi:10.1186/s12862-017-0904-4)
Supplement: Additional file 4: Figure S1. — SDS-PAGE of reduced G. tridactyla venom. Figure S2. Genomic organization of the glycerotoxin gene analyzed in G. tridactyla. Figure S3. Immunolocalization of GLTx in a cross section through a putative venom gland embedded in paraffin. Figure S4. Anti-serotonin (5-HT) staining and phalloidin–rhodamine counterstaining on everted G. tridactyla pharynges cut into two halves. Figure S5. Quantitative real-time PCR (qPCR) expression levels (shown as Fold Change, RQ) between biological groups (putative venom glands [A], pharyngeal lobes [B], and posterior body wall [C]) per analyzed specimen (n = 10, biological samples). Figure S6. Tissues analyzed in comparative GLTx expression studies (qPCR experiments and transcriptome analyses) on G. tridactyla. (PDF 2931 kb) [file 12862_2017_904_MOESM4_ESM.pdf]

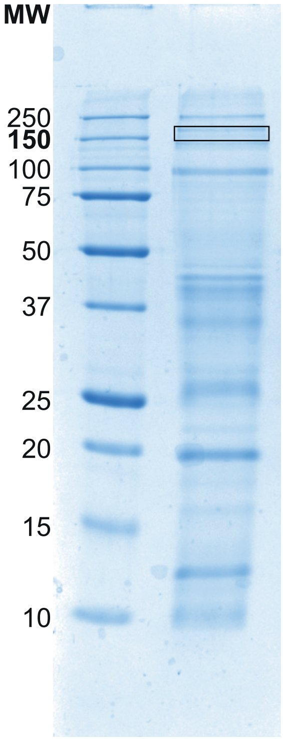

Figure S1. SDS-PAGE of reduced *G. tridactyla* venom. In-gel digestion and LC-MS/MS analyses identified GLTx in two almost identical bands corresponding to a mass of approximately 150 kDa (highlighted in dashed box, see also Additional file 5). This matches the predicted mass from the amino acid sequence of GLTx, and suggests it exists as a disulfide-bridged dimer in its native state. MW, molecular weight; marker, Precision Plus Protein Prestained Standard (Bio-Rad)

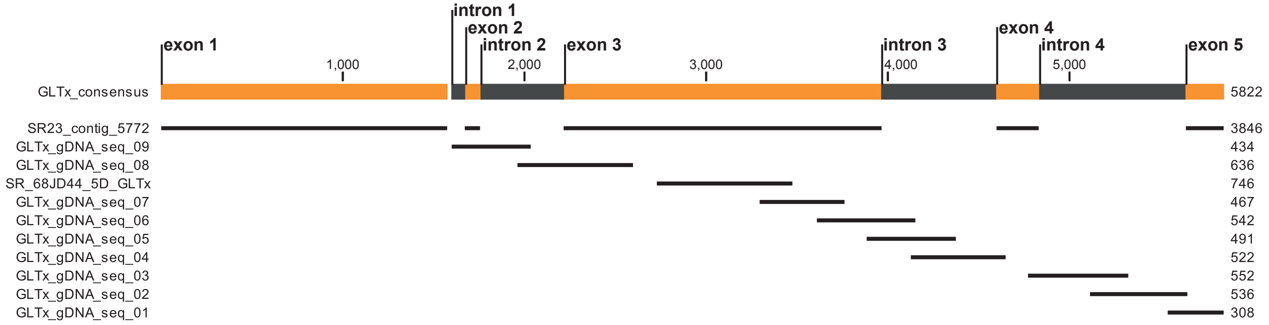

Figure S2. Genomic organization of the glycerotoxin gene analyzed in *G. tridactyla*. GLTx shows an intron-exon-structure. Introns are marked in grey, exons in orange. Analysis focused on the intron-exon-structure at the 3'-end, whereas it remains unknown at the 5'-end (exon 1) of the GLTx gene

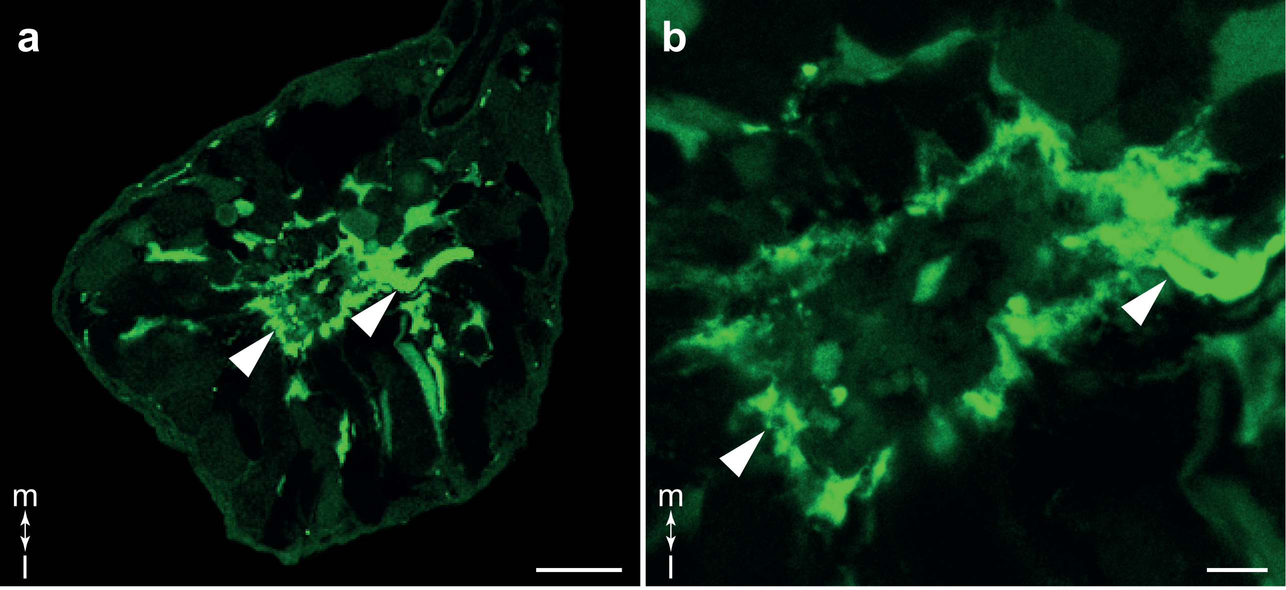

Figure S3. Immunolocalization of GLTx in adult *G. tridactyla*. Confocal laser scanning micrographs. Arrows indicate GLTx-IR staining. **a–b** Cross section through a putative venom gland embedded in paraffin. Distinct GLTx-IR staining is detectable inside the lumen of the putative venom gland. Scale bars: 50 µm (**a**), and 10 µm (**b**)

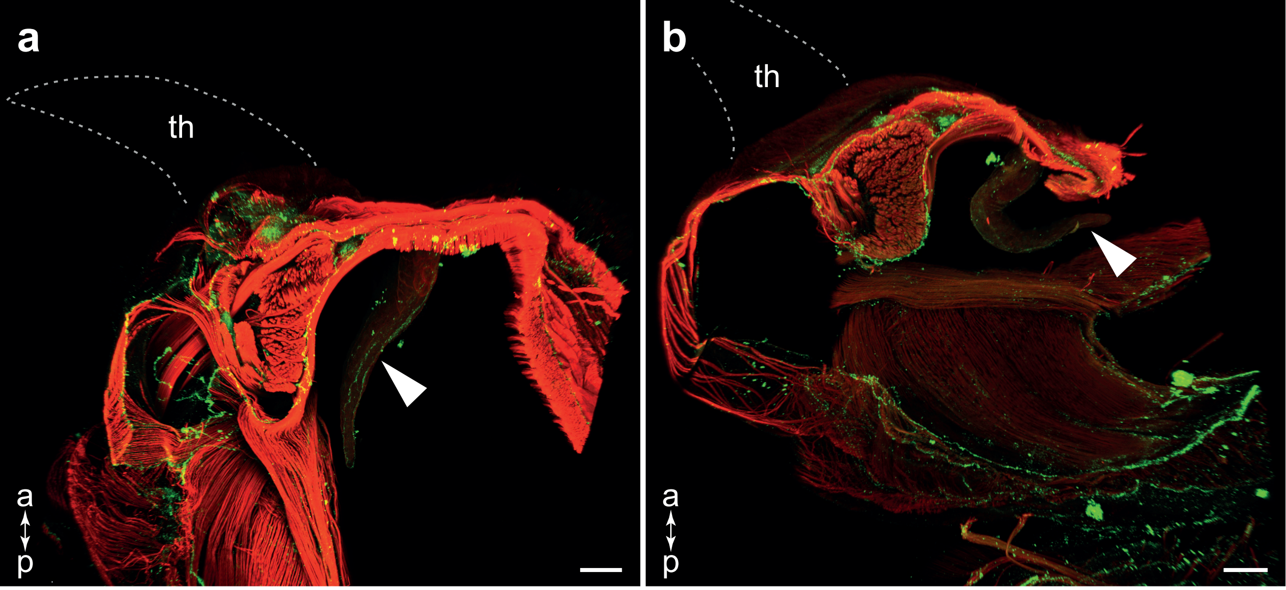

Figure S4. Confocal maximum projections of everted *G. tridactyla* pharynges cut into two halves. Arrows indicate position of the pharyngeal lobes. **a–b** Anti-serotonin (5-HT) staining (green) and phalloidin–rhodamine counterstaining (red). 5-HT-IR staining revealed neural innervation of the lobe, but absence of prominent somata clusters or neuropils therein. Phalloidin staining exhibits dense muscle bundles within the entire pharynx lacking the lobes. th, teeth. Scale bars: 100 µm (**a–b**)

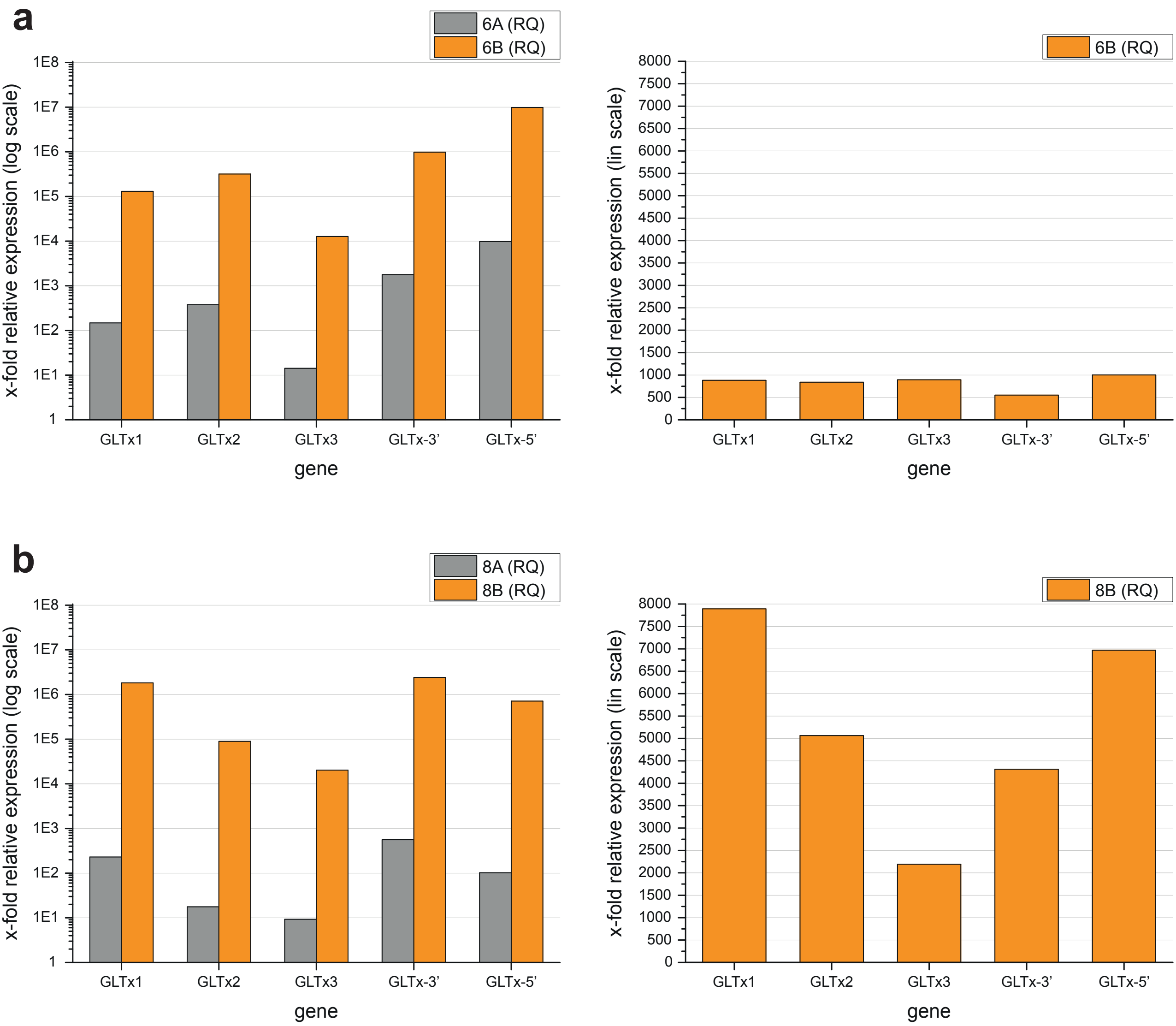

Figure S5. Quantitative real-time PCR (qPCR) expression levels (shown as Fold Change, RQ) between biological groups (putative venom glands [A], pharyngeal lobes [B], and posterior body wall [C]) per analyzed specimen (n=10, biological samples)

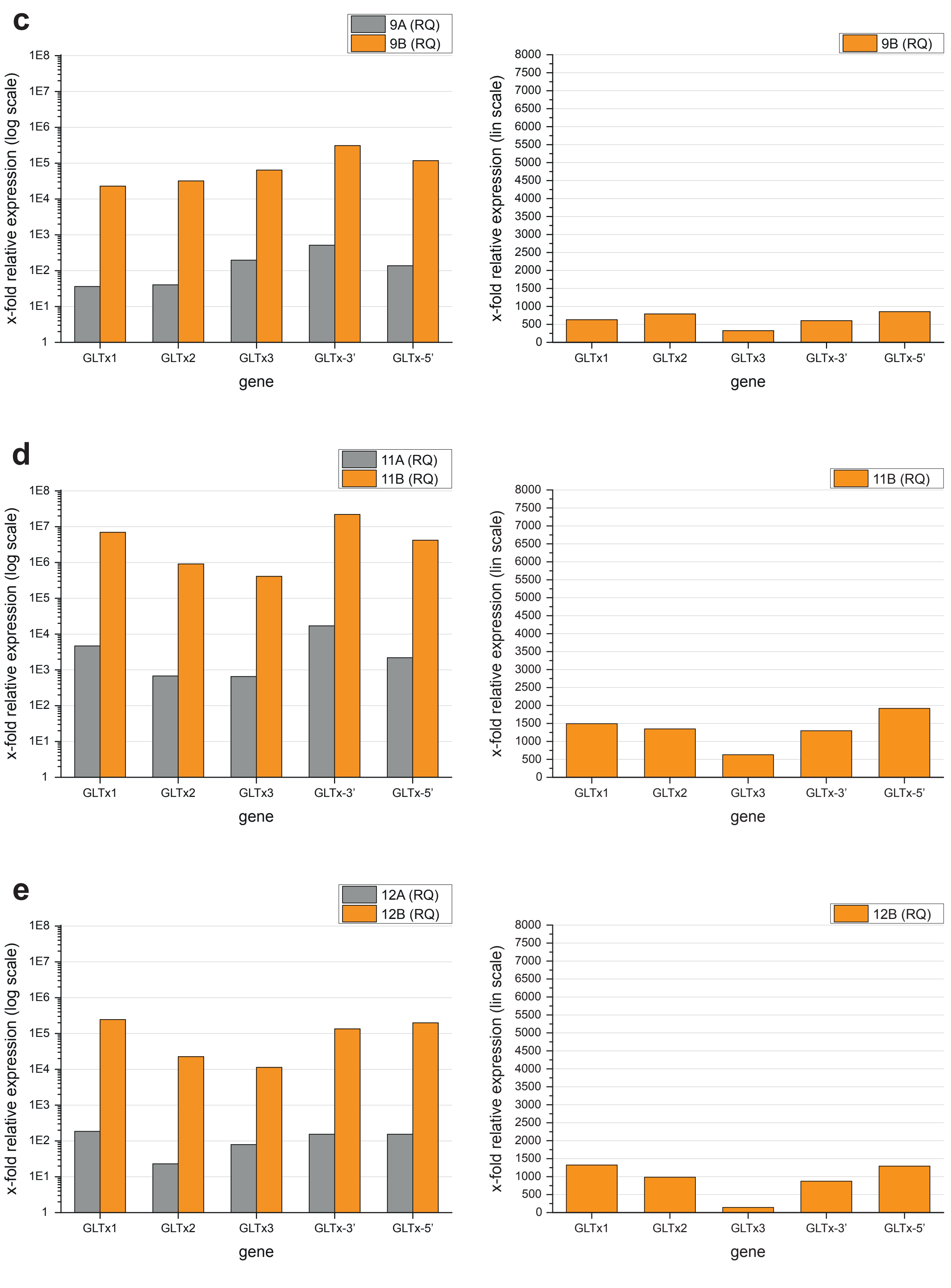

Figure S5. (continue)

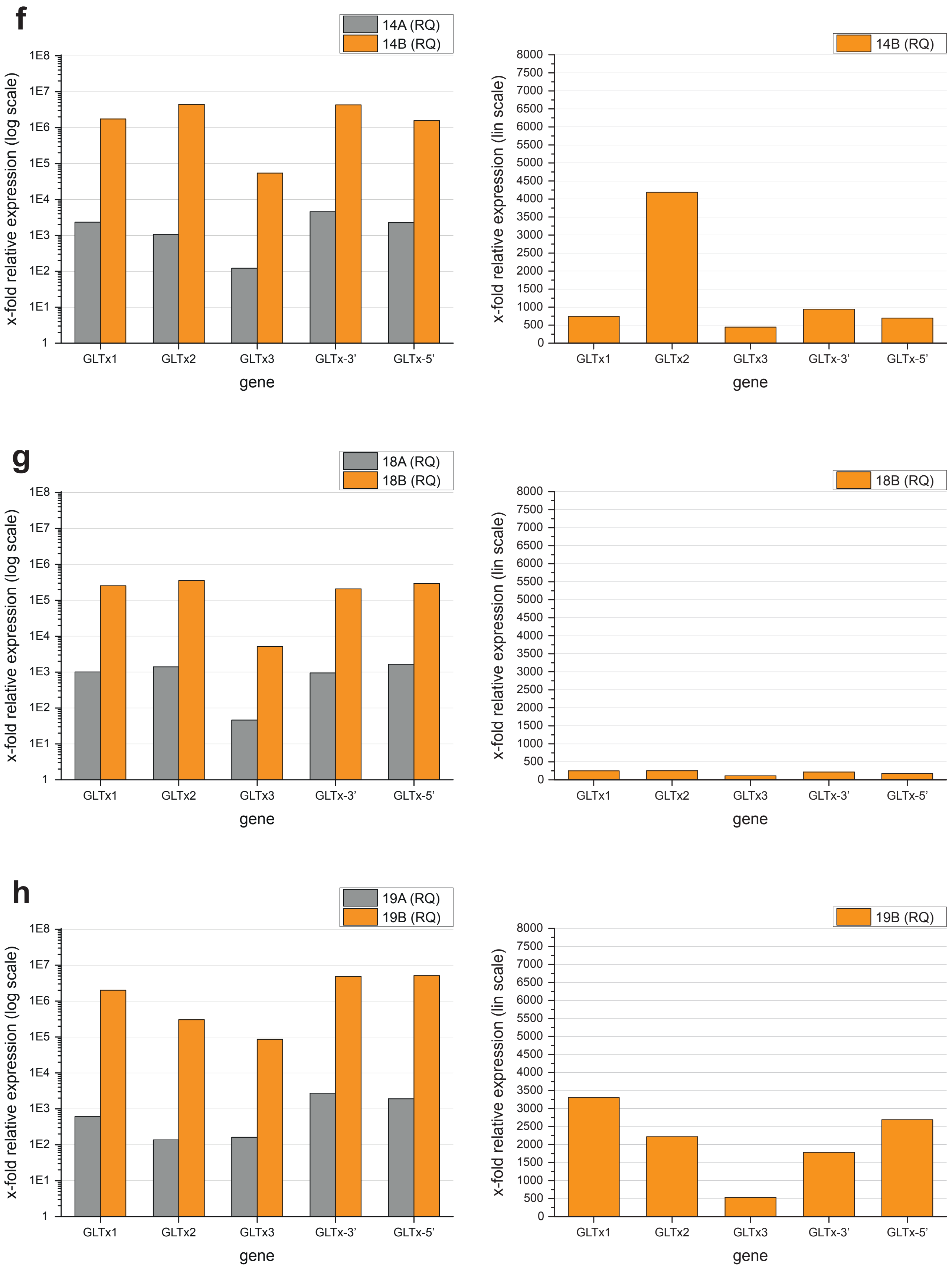

Figure S5. (continue)

i

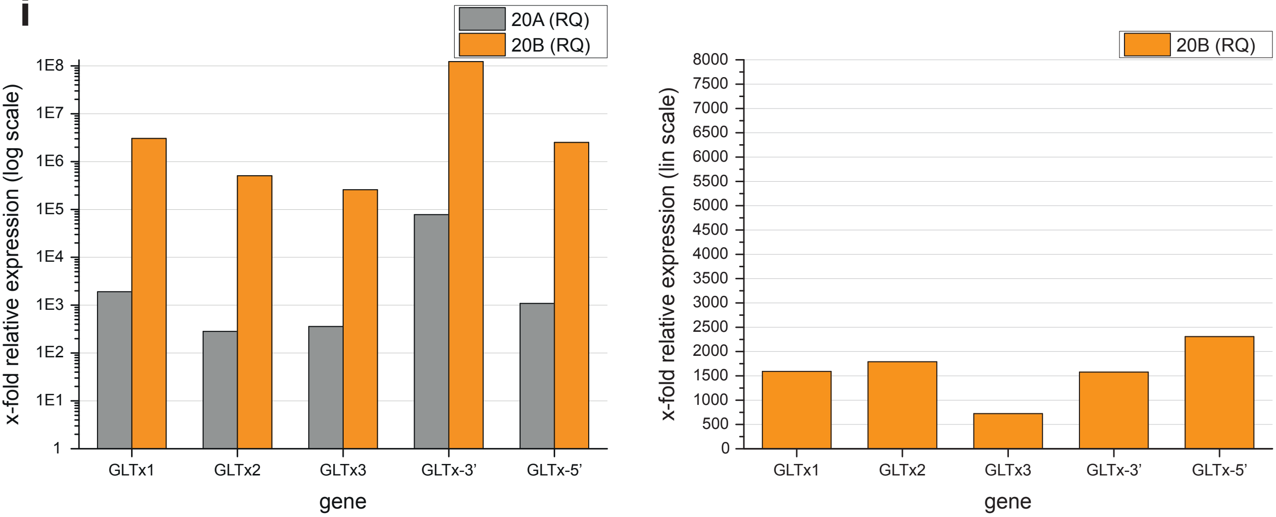

j

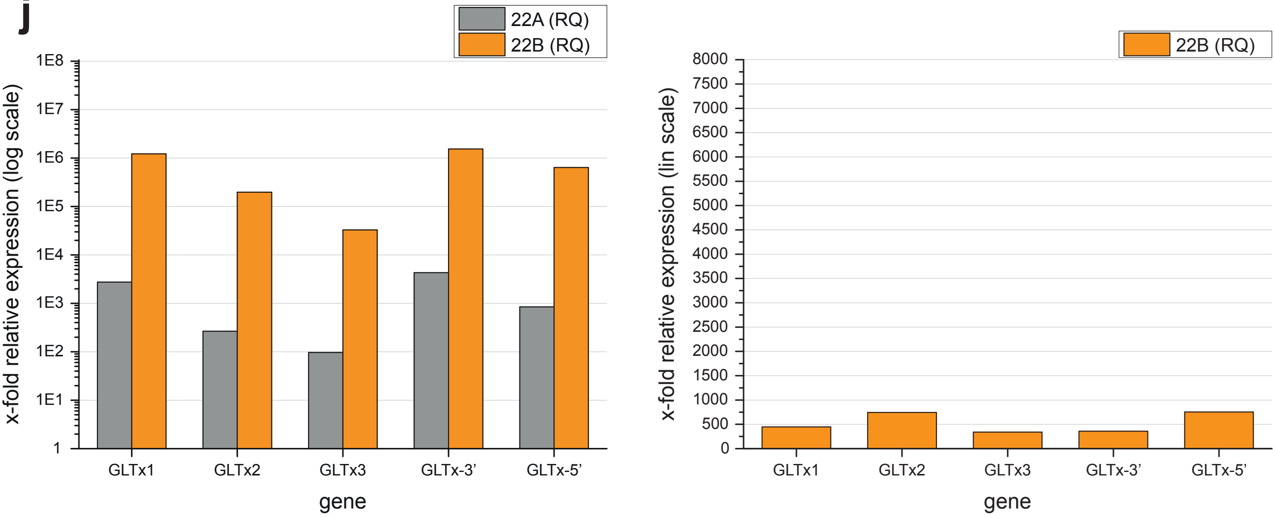

Figure S5. (continue)

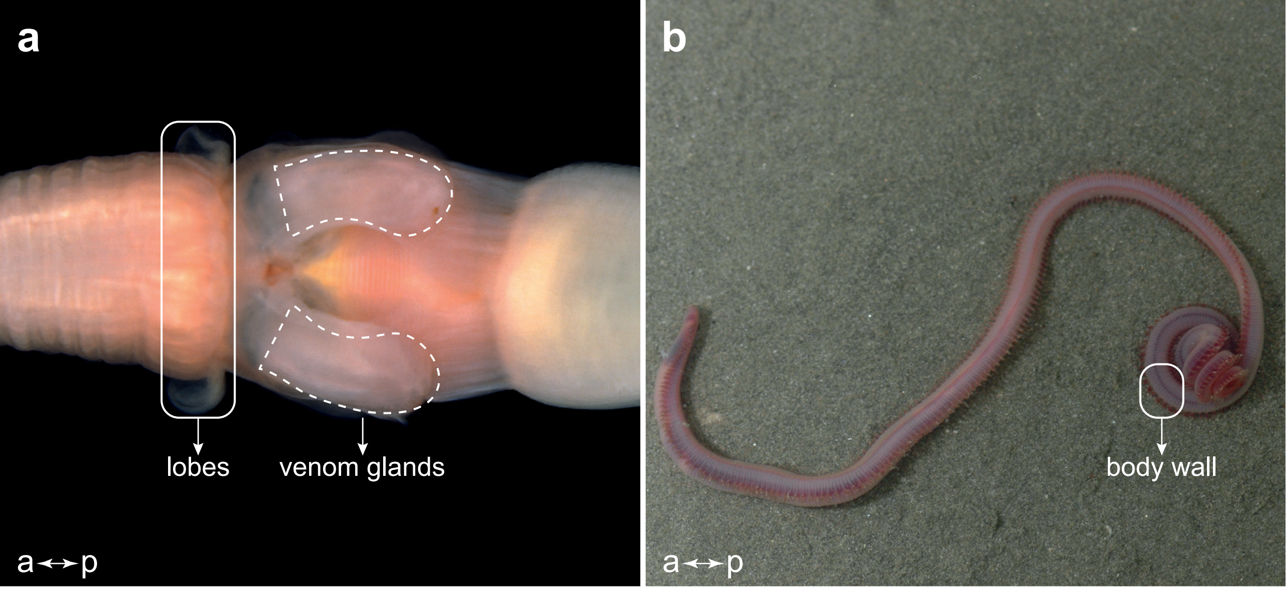

Figure S6. Tissues analyzed in comparative GLTx expression studies (qPCR experiments and transcriptome analyses) on *G. tridactyla*. The relative GLTx expression was determined in putative venom glands and pharyngeal lobes (**a**), as well as in the posterior body wall (**b**)
