# Supplementary material for: Comparative analyses of glycerotoxin expression unveil a novel structural organization of the bloodworm venom system
Source: BMC Evol Biol. 2017 Mar 4;17:64. doi: 10.1186/s12862-017-0904-4 (PMC5336659; doi:10.1186/s12862-017-0904-4)
Supplement: Additional file 13: — Protocol S1–S3. Protocol S1. Protocol: In situ hybridization on pharynx tissue of G. tridactyla (Glyceridae, Annelida). Protocol S2. Protocol: Anti-GLTx staining on pharynx tissue of G. tridactyla (Glyceridae, Annelida). Protocol S3. Protocol: Fluorescence in situ hybridization (FISH) coupled with antibody staining against GLTx on pharynx tissue of G. tridactyla. (PDF 590 kb) [file 12862_2017_904_MOESM13_ESM.pdf]

Protocol S1.

PROTOCOL

In situ hybridization on pharynx tissue of *Glycera tridactyla* (Glyceridae, Annelida)

Richter *et al*. *BMC Evol Biol*. 2017

DATE	TISSUE	SAMPLE NAME	NOTES

LEGEND

⇨ ***ATTENTION*** ❖ ***HINT*** ✋ ***REST***

DAY 1

Rehydration & Digestion

⇨ all steps were performed at room temperature,
incubation for 5 min each, on a shaker

❖ prepare a serial dilution of a methanol/PTW mixture in a 6-well plate

and stepwise rehydrate samples in:

☐ 100% methanol

☐ 60% methanol/40% PTW

☐ 30% methanol/70% PTW

☐☐☐☐ 4 x washing in 100% PTW

☐ digestion through proteinase K (0.01 mg/ml in PTW)

⇨ incubation for 5 min, but without any shaking

☐☐ stop digestion through 2 x washing in glycine/PTW (2 mg/ml)

☐ 1x washing in 1% triethanolamine in PTW

- ☐ to permeabilize the cells add glacial acetic acid, 5 min incubation
- ☐ add again glacial acetic acid, 5 min incubation

☐☐ 2 x washing in 100% PTW

☐ re-fixation in 4% paraformaldehyde in PTW

⇨ incubation for 60 min on a shaker

☐☐☐☐☐ 5 x washing in PTW

☐ transfer the pharynx samples in new 2 ml non-sticky tubes filled with PTW, incubation for 5 min on a shaker

☐ ⇨ heat samples to 80 °C for 10 min, in heating block, without shaking

Pre-hybridization

☐ remove liquids completely and add hybridization buffer

⇨ incubation for 10 min at room temperature

☐ remove liquids completely and add 65 °C pre-warmed hybridization buffer

✋ incubation overnight at 65 °C, in water bath

DAY 2 (3/4)

Hybridization

❖ dilute digoxigenin-labeled RNA probes (SP6/T7) in hybridization buffer, concentration: 1 ng/µl in hybridization buffer,
(for probe construction see methods section in the main text)

⇨ per pharynx: one half will be treated with probe SP6, the other half with probe T7
(refers to sense and antisense/reaction and negative control)

☐ denaturation of diluted probes through heating to 80 °C for 10 min in a heating block

⇨ without shaking

☐ transfer of pharynx samples in tubes containing the to 80 °C heated probes

incubation for 72 h at 65 °C, in water bath

❖ RNA probe will hybridize to the corresponding mRNA

DAY 5

Washing

⇨ all required reagents are pre-warmed to 65 °C

⇨ incubation steps are performed in a water bath at 65 °C

☐ remove liquids completely

☐ 1 x washing in hybridization buffer, incubation for 5 min

☐ 1 x washing in hybridization buffer, incubation for 20 min

⇨ incubation steps are performed in a water bath at 65 °C, incubation for 10 min each

❖ prepare a serial dilution of hybridization buffer/2 x SSC

☐ 1 x washing in 75% hybridization buffer/25% 2 x SSC

☐ 1 x washing in 50% hybridization buffer/50% 2 x SSC

☐ 1 x washing in 25% hybridization buffer/75% 2 x SSC

☐ 1 x washing in 100% 2 x SSC,
to remove excess probe and hybridization buffer

☐☐ ⇨ 2 x washing for 30 min each in 0.02 x SSC,
to remove non-specific RNA hybridization

⇨ incubation steps are performed at room temperature,
incubation for 5 min each, on a shaker

❖ prepare a serial dilution of 0.02 x SSC/PTW

☐ 1 x washing in 75% 0.02 x SSC/25% PTW

☐ 1 x washing in 50% 0.02 x SSC/50% PTW

☐ 1 x washing in 25% 0.02 x SSC/75% PTW

☐ 1 x washing in 100% PTW

☐☐☐☐☐ 5 x washing in PTW

Visualization

⇨ steps are performed on a shaker

☐ blocking in blocking buffer (5% normal goat serum in PTW),
for 1 h at room temperature

☐ incubation with the anti-digoxigenin antibody (Roche, #11093274910),
diluted at 1:5000 in blocking buffer
(2.5 % normal goat serum in PTW + 1 µl Anti-Digoxigenin-AP, Fab fragments [150 U])

✋ incubation overnight at 4 °C

DAY 6

Color Staining

⇨ steps are performed at room temperature, on a shaker

☐☐☐☐
☐☐☐☐ 8 x washing in PTW, incubation for 10 min each

☐ to remove PTW completely, transfer the pharynx samples in new tubes

❖ excess PTW will precipitate with AP staining buffer

☐☐☐ 3 x washing in AP staining buffer, incubation for 5 min each

☐ color staining in NBT/BCIP staining solution
(6.6 µl NBT/ml and 3.3 µl BCIP/ml in AP staining buffer)

⇨ light-sensitive, at room temperature, without shaking

❖ weak signal after 15 min,
strong signal after 45 min–3 h (tissue and species specific)

☐ stop staining reaction by adding 4% paraformaldehyde in PTW, incubation for 1 h at room temperature, on a shaker

⇨ keep samples in the dark

☐ 1 x washing in PTW

⇨ keep samples in the dark

✋ overnight at 4 °C, on a shaker

DAY 7

Storage

☐☐ 2 x washing in PTW, incubation for 2 h each at 4 °C, on a shaker

⇨ keep samples in the dark

☐ storage until imaging:

⇨ in the dark at 4 °C, without shaking

☐ storage after imaging: transfer samples in solution of 0.1 M PBS containing 0.005% sodium azide

⇨ in the dark at 4 °C, without shaking

BUFFER STOCKS

<u>10 x PBS (100 ml), pH 7.4</u>

dissolve	8.0 g NaCl
	0.2 g KCl
	0.24 g KH_2PO_4
	1.44 g Na_2HPO_4 x 2 H_2O
in	90 ml ddH_2O
adjust to	pH 7.4
and fill up to	100 ml with ddH_2O
mix and autoclave	

<u>20 x SSC (1 l), pH 7.0</u>

dissolve	88.2 g 0.3 M Na-Citrat
	175.3 g 3 M NaCl
in	990 ml ddH_2O
adjust to	pH 7.0
and fill up to	1 l with ddH_2O
mix and autoclave	

<u>1 x PTW (500 ml)</u>

1 x PBS + 0.1% Tween-20

mix	50 ml 10 x PBS + 500 µl Tween-20
and fill up to	500 ml with autoclaved ddH_2O
not autoclave	

<u>0.1 M PBS (1 l), pH 7.4</u>

dissolve	5.0 g NaCl
	14.0 g Na_2HPO_4 x 2H_2O
	3.2 g NaH_2PO_4 x H_2O
in	990 ml ddH_2O
adjust to	pH 7.4
and fill up to	1 l with ddH_2O

Hybridization buffer

not autoclave
premake, aliquot and store at –20C

chemical	stock	amount for 50 ml	amount for 200 ml
Formamide	100%	25 ml	100 ml
SSC	20 x	12.5 ml	50 ml
Heparin	50 mg/ml<br>200 mg/ml	150 µl<br>37.5 µl	600 µl<br>150 µl
Torula-RNA	solid	250 mg	1 g
Tween-20	10%<br>100%	500 µl<br>50 µl	2 ml<br>200 µl
ddH_2O		adjust to 50 ml	adjust to 200 ml

AP staining buffer, pH 9.5

not autoclave, prepare just prior to use
adjust to pH 9.5
store at 4 °C

chemical	stock	amount for 50 ml	amount for 200 ml
TrisCl, pH 9.5	2 M	2.5 ml	10 ml
NaCl	5 M	1.0 ml	4.0 ml
$MgCl_2$	1 M	2.5 ml	10 ml
Tween-20	100%	50 µl	200 µl
ddH_2O		adjust to 50 ml	adjust to 200 ml

Protocol S2.

PROTOCOL

Anti-GLTx staining on pharynx tissue of *Glycera tridactyla* (Glyceridae, Annelida)

Richter *et al*. *BMC Evol Biol*. 2017

DATE	TISSUE	SAMPLE NAME	NOTES

LEGEND

⇨ ***ATTENTION*** ❖ ***HINT*** ✋ ***REST***

DAY 1

Tissue permeabilization

⇨ everted pharynx was dissected into two halves

❖ add the following reaction mixture to the tissue samples:

☐ 940 µl 0.1 M PBS

☐ 60 µl block-PTA (6% normal goat serum in block-PTA)

☐ 5 µl 0.1% Triton X-100 (PTA)

☐ 1 µl 0.1% sodium azide (NaN_3)

☐ ✋ incubation overnight at room temperature, on a shaker

DAY 2 (3/4)

Primary antibody

discard liquids and continue working in the same block dishes

add the following reaction mixture to the tissue samples:

☐ 940 µl 0.1 M PBS

☐ 60 µl block-PTA (6% normal goat serum in block-PTA)

☐ 5 µl 0.1% Triton X-100 (PTA)

☐ 1 µl 0.1% sodium azide (NaN_3)

☐ primary antibody monoclonal mouse anti-GLTx (4G9, Meunier *et al*. *EMBO J*. 2002), diluted 1:500

☐ incubation for 72 h at 4 °C, on a shaker

DAY 5 (6/7)

Washing

3 x washing for 2 h at room temperature, on a shaker

discard liquids each time and continue working in the same block dishes

add the following reaction mixture to the tissue samples:

☐ 940 µl 0.1 M PBS

☐ 60 µl block-PTA (6% normal goat serum in block-PTA)

☐ 5 µl 0.1% Triton X-100 (PTA)

☐ incubation for 2 h at room temperature, on a shaker

❖ add the following reaction mixture to the tissue samples:

☒☐ 940 µl 0.1 M PBS

☒☐ 60 µl block-PTA (6% normal goat serum in block-PTA)

☒☐ 5 µl 0.1% Triton X-100 (PTA)

☒☐ incubation for 2 h at room temperature, on a shaker

❖ add the following reaction mixture to the tissue samples:

☒☒☐ 940 µl 0.1 M PBS

☒☒☐ 60 µl block-PTA (6% normal goat serum in block-PTA)

☒☒☐ 5 µl 0.1% Triton X-100 (PTA)

☒☒☐ incubation for 2 h at room temperature, on a shaker

Secondary fluorochrome conjugated antibody

⇨ discard liquids and continue working in the same block dishes

❖ add the following reaction mixture to the tissue samples:

☐ 940 µl 0.1 M PBS

☐ 60 µl block-PTA (6% normal goat serum in block-PTA)

☐ 5 µl 0.1% Triton X-100 (PTA)

☐ 1 µl 0.1% sodium azide (NaN_3)

☐ secondary fluorochrome conjugated antibody (goat anti-mouse Alexa Fluor 488 (Invitrogen, Carlsbad, CA, USA), diluted 1:500

☐ incubation for 72 h at 4 °C, on a shaker

⇨ light-sensitive

DAY 8

⇨ discard liquids and continue working in the same block dishes

⇨ keep samples in the dark

Washing

⇨ steps were performed at room temperature, on a shaker

☐☐ 2 x washing in 1ml 0.1 M PBS, incubation for 1.5 h–2 h

☐ incubation for 2 h in 1 ml 0.1 M PBS + 10 µl phalloidin–rhodamine from methanolic stock solution (Invitrogen, Darmstadt, Germany)

Dehydration and Embedding

⇨ steps were performed at room temperature, without shaking, incubation for 5 min each

☐ 1 x washing in 70% isopropanol

☐ 1 x washing in 85% isopropanol

☐ 1 x washing in 95% isopropanol

☐☐ 2 x washing in 100% isopropanol

⇨ steps were performed at room temperature, without shaking

☐ remove liquids completely

☐ incubation for 10 min in Murray's clearing solution

☐ remove liquids completely

☐ mount between two coverslips in DPX

Storage

☐ store samples in the dark at 4 °C, without shaking

BUFFER STOCKS

0.1 M PBS (1 l), pH 7.4

dissolve	5.0 g NaCl
	14.0 g Na_2HPO_4 x $2H_2O$
	3.2 g NaH_2PO_4 x H_2O
in	990 ml ddH_2O
adjust to	pH 7.4
and fill up to	1 l with ddH_2O

Murray's clearing solution

mix	benzyl alcohol
	benzyl benzoate
at a ratio of	1:2

Protocol S3.

PROTOCOL

Fluorescence *in situ* hybridization (FISH) coupled with antibody staining against GLTx on pharynx tissue of *Glycera tridactyla*

Richter *et al*. *BMC Evol Biol*. 2017

DATE	TISSUE	SAMPLE NAME	NOTES

LEGEND

⇨ ***ATTENTION*** ❖ ***HINT*** ✋ ***REST***

DAY 1

Rehydration & Digestion

⇨ all steps were performed at room temperature,
incubation for 5 min each, on a shaker

❖ prepare a serial dilution of a methanol/PTW mixture in a 6-well plate and stepwise rehydrate samples in:

- ☐ 100% methanol
- ☐ 60% methanol/40% PTW
- ☐ 30% methanol/70% PTW

☐☐☐☐ 4 x washing in 100% PTW

☐ digestion through proteinase K (0.01 mg/ml in PTW)

⇨ incubation for 5 min, but without any shaking

☐☐ stop digestion through 2 x washing in glycine/PTW (2 mg/ml)

☐ 1x washing in 1% triethanolamine in PTW

- ☐ to permeabilize the cells add glacial acetic acid, 5 min incubation
- ☐ add again glacial acetic acid, 5 min incubation

☐☐ 2 x washing in 100% PTW

☐ re-fixation in 4% paraformaldehyde in PTW

⇨ incubation for 60 min on a shaker

☐☐☐☐☐ 5 x washing in PTW

☐ transfer the pharynx samples in new 2 ml non-sticky tubes filled with PTW, incubation for 5 min on a shaker

☐ ⇨ heat samples to 80 °C for 10 min, in heating block, without shaking

Pre-hybridization

☐ remove liquids completely and add hybridization buffer

⇨ incubation for 10 min at room temperature

☐ remove liquids completely and add 65 °C pre-warmed hybridization buffer

🖐 incubation overnight at 65 °C, in water bath

DAY 2 (3/4)

Hybridization

❖ dilute digoxigenin-labeled RNA probes (SP6/T7) in hybridization buffer, concentration: 1 ng/µl in hybridization buffer,
(for probe construction see methods section in the main text)

☐ denaturation of diluted probes through heating to 80 °C for 10 min in a heating block

⇨ without shaking

☐ transfer of pharynx samples in tubes containing the to 80 °C heated probes

✋ incubation for 72 h at 65 °C, in water bath

❖ RNA probe will hybridize to the corresponding mRNA

DAY 5

Washing

⇨ all required reagents are pre-warmed to 65 °C

⇨ incubation steps are performed in a water bath at 65 °C

☐ remove liquids completely

☐ 1 x washing in hybridization buffer, incubation for 5 min

☐ 1 x washing in hybridization buffer, incubation for 20 min

⇨ incubation steps are performed in a water bath at 65 °C, incubation for 10 min each

❖ prepare a serial dilution of hybridization buffer/2 x SSC

☐ 1 x washing in 75% hybridization buffer/25% 2 x SSC

☐ 1 x washing in 50% hybridization buffer/50% 2 x SSC

☐ 1 x washing in 25% hybridization buffer/75% 2 x SSC

☐ 1 x washing in 100% 2 x SSC,
to remove excess probe and hybridization buffer

☐☐ ⇨ 2 x washing for 30 min each in 0.02 x SSC,
to remove non-specific RNA hybridization

⇨ start working in black block dishes

⇨ incubation steps are performed at room temperature, incubation for 5 min each, on a shaker

❖ prepare a serial dilution of 0.02 x SSC/TNT

☐ 1 x washing in 75% 0.02 x SSC/25% TNT

☐ 1 x washing in 50% 0.02 x SSC/50% TNT

☐ 1 x washing in 25% 0.02 x SSC/75% TNT

☐ 1 x washing in 100% TNT

☐☐☐☐☐ 5 x washing in TNT

Visualization

⇨ steps are performed on a shaker

☐ blocking in TNB blocking buffer (0.5% blocking reagent, PerkinElmer, #FP1012), for 3 h at room temperature

☐ incubation with the anti-digoxigenin antibody (Roche, #11207733910), diluted at 1:100 in TNB blocking buffer
(1 ml TNB + 10 µl Anti-Digoxigenin-POD, Fab fragments [150 U])

✋ incubation overnight at 4 °C

DAY 6

Color Staining

⇨ steps are performed at room temperature

☐☐☐☐☐ 5 x washing in TNT, incubation for 10 min each, on a shaker

☐ color staining in FITC-Tyramide staining solution (TSA™ Plus Fluorescein System, PerkinElmer, #NEL741001KT), diluted at 1:50 in 1 x Plus Amplification Diluent (12 µl FITC-Tyramide + 588 µl 1 x Plus Amplification Diluent)

⇨ light-sensitive, at room temperature, without shaking

❖ strong signal after 30 min

⇨ from here beginning, keep samples always in the dark

☐ 1 x washing in TNT to stop staining reaction, incubation for 5 min at room temperature, on a shaker

☐ transfer samples in new black block dishes

☐ 1 x washing in TNT, incubation for 10 min at room temperature, on a shaker

⇨ incubation steps are performed at room temperature, incubation for 5 min each, on a shaker

❖ prepare a serial dilution of TNT/0.1 M PBS

☐ 1 x washing in 75% TNT/25% 0.1 M PBS

☐ 1 x washing in 50% TNT/50% 0.1 M PBS

☐ 1 x washing in 25% TNT/75% 0.1 M PBS

☐☐☐ 3 x washing in 100% 0.1 M PBS

Anti-GLTx staining, Primary antibody

⇨ continue working in the same black block dishes

❖ add the following reaction mixture to the tissue samples:

☐ 940 µl 0.1 M PBS

☐ 60 µl block-PTA (6% normal goat serum in block-PTA)

☐ 5 µl 0.1% Triton X-100 (PTA)

☐ ✋ incubation for 30 min at room temperature, on a shaker

⇨ discard liquids and continue working in the same block dishes

❖ add the following reaction mixture to the tissue samples:

☐ 940 µl 0.1 M PBS

☐ 60 µl block-PTA (6% normal goat serum in block-PTA)

☐ 5 µl 0.1% Triton X-100 (PTA)

☐ 1 µl 0.1% sodium azide (NaN_3)

☐ primary antibody monoclonal mouse anti-GLTx (4G9, Meunier *et al*. *EMBO J*. 2002), diluted 1:500

☐ incubation overnight at 4 °C, on a shaker

DAY 7

Washing

⇨ 3 x washing for 2 h at room temperature, on a shaker

⇨ discard liquids each time and continue working in the same block dishes

❖ add the following reaction mixture to the tissue samples:

☐ 940 µl 0.1 M PBS

☐ 60 µl block-PTA (6% normal goat serum in block-PTA)

☐ 5 µl 0.1% Triton X-100 (PTA)

☐ incubation for 2 h at room temperature, on a shaker

❖ add the following reaction mixture to the tissue samples:

☒☐ 940 µl 0.1 M PBS

☒☐ 60 µl block-PTA (6% normal goat serum in block-PTA)

☒☐ 5 µl 0.1% Triton X-100 (PTA)

☒☐ incubation for 2 h at room temperature, on a shaker

❖ add the following reaction mixture to the tissue samples:

☒☒☐ 940 µl 0.1 M PBS

☒☒☐ 60 µl block-PTA (6% normal goat serum in block-PTA)

☒☒☐ 5 µl 0.1% Triton X-100 (PTA)

☒☒☐ incubation for 2 h at room temperature, on a shaker

Anti-GLTx staining, Secondary fluorochrome conjugated antibody

⇨ discard liquids and continue working in the same block dishes

❖ add the following reaction mixture to the tissue samples:

☐ 940 µl 0.1 M PBS

☐ 60 µl block-PTA (6% normal goat serum in block-PTA)

☐ 5 µl 0.1% Triton X-100 (PTA)

☐ 1 µl 0.1% sodium azide (NaN_3)

☐ secondary fluorochrome conjugated antibody (goat anti-mouse Alexa Fluor 568 (Invitrogen, Carlsbad, CA, USA), diluted 1:500

☐ incubation overnight at 4 °C, on a shaker

⇨ light-sensitive

DAY 8

⇨ discard liquids and continue working in the same block dishes

⇨ keep samples in the dark

Washing

⇨ steps were performed at room temperature, on a shaker

☐☐ 2 x washing in 1ml 0.1 M PBS, incubation for 1 h each

☐ incubation for 1 h in 600 µl 0.1 M PBS + 1.5 µl TO-PRO®-3 Iodide (Life Technologies, #T3605)

Dehydration and Embedding

⇨ steps were performed at room temperature, without shaking, incubation for 5 min each

☐ 1 x washing in 70% isopropanol

☐ 1 x washing in 85% isopropanol

☐ 1 x washing in 95% isopropanol

☐☐ 2 x washing in 100% isopropanol

⇨ steps were performed at room temperature, without shaking

☐ remove liquids completely

☐ incubation for 10 min in Murray’s clearing solution

☐ remove liquids completely

☐ mount between two coverslips in DPX

Storage

☐ store samples in the dark at 4 °C, without shaking

BUFFER STOCKS

10 x PBS (100 ml), pH 7.4

dissolve	8.0 g NaCl
	0.2 g KCl
	0.24 g KH_2PO_4
	1.44 g Na_2HPO_4 x 2 H_2O
in	90 ml ddH_2O
adjust to	pH 7.4
and fill up to	100 ml with ddH_2O
mix and autoclave	

20 x SSC (1 l), pH 7.0

dissolve	88.2 g 0.3 M Na-Citrat
	175.3 g 3 M NaCl
in	990 ml ddH_2O
adjust to	pH 7.0
and fill up to	1 l with ddH_2O
mix and autoclave	

1 x PTW (500 ml)

1 x PBS + 0.1% Tween-20

mix	50 ml 10 x PBS + 500 µl Tween-20
and fill up to	500 ml with autoclaved ddH_2O
not autoclave	

10 x TN (100 ml), pH 7.5

dissolve	12.11 g Tris-base (1 M Tris-HCl)
	8.76 g 1.5 M NaCl
in	80 ml ddH2O
adjust to	pH 7.5
and fill up to	100 ml with ddH_2O
mix and autoclave	

1 x TNT (200 ml)

1 x TN + 0.1% Tween-20

mix	20 ml 10 x TN + 200 µl Tween-20
and fill up to	200 ml with autoclaved ddH_2O
not autoclave	

1 x TNB (100 ml)

mix	1 x TNT + 0.5% blocking reagent (PerkinElmer, #FP1012)

dissolve	0.5 g blocking reagent
in	100 ml 1 x TNT
heat to	60 °C while stirring
not autoclave	
aliquot and store at -20 °C	

0.1 M PBS (1 l), pH 7.4

dissolve	5.0 g NaCl
	14.0 g Na_2HPO_4 x $2H_2O$
	3.2 g NaH_2PO_4 x H_2O
in	990 ml ddH_2O
adjust to	pH 7.4
and fill up to	1 l with ddH_2O

Hybridization buffer

not autoclave
premake, aliquot and store at –20C

chemical	stock	amount for 50 ml	amount for 200 ml
Formamide	100%	25 ml	100 ml
SSC	20 x	12.5 ml	50 ml
Heparin	50 mg/ml<br>200 mg/ml	150 µl<br>37.5 µl	600 µl<br>150 µl
Torula-RNA	solid	250 mg	1 g
Tween-20	10%<br>100%	500 µl<br>50 µl	2 ml<br>200 µl
ddH_2O		adjust to 50 ml	adjust to 200 ml
